# Complete biosynthesis of QS-21 in engineered yeast

**DOI:** 10.1038/s41586-024-07345-9

**Published:** 2024-05-08

**Authors:** Yuzhong Liu, Xixi Zhao, Fei Gan, Xiaoyue Chen, Kai Deng, Samantha A. Crowe, Graham A. Hudson, Michael S. Belcher, Matthias Schmidt, Maria C. T. Astolfi, Suzanne M. Kosina, Bo Pang, Minglong Shao, Jing Yin, Sasilada Sirirungruang, Anthony T. Iavarone, James Reed, Laetitia B. B. Martin, Amr El-Demerdash, Shingo Kikuchi, Rajesh Chandra Misra, Xiaomeng Liang, Michael J. Cronce, Xiulai Chen, Chunjun Zhan, Ramu Kakumanu, Edward E. K. Baidoo, Yan Chen, Christopher J. Petzold, Trent R. Northen, Anne Osbourn, Henrik Scheller, Jay D. Keasling

**Affiliations:** 1 California Institute of Quantitative Biosciences (QB3), University of California, Berkeley, Berkeley, CA USA; 2 Joint BioEnergy Institute, Emeryville, CA USA; 3 Department of Plant and Microbial Biology, University of California, Berkeley, Berkeley, CA USA; 4 Environmental Genomics and Systems Biology Division, Lawrence Berkeley National Laboratory, Berkeley, CA USA; 5 Department of Biomaterials and Biomanufacturing, Sandia National Laboratories, Livermore, CA USA; 6 Department of Chemical and Biomolecular Engineering, University of California, Berkeley, Berkeley, CA USA; 7 Division of Biological Systems and Engineering, Lawrence Berkeley National Laboratory, Berkeley, CA USA; 8 Institute of Applied Microbiology, Aachen Biology and Biotechnology, RWTH Aachen University, Aachen, Germany; 9 Department of Bioengineering, University of California, Berkeley, Berkeley, CA USA; 10 Center for Biomolecular Structure, Function and Application, Suranaree University of Technology, Nakhon Ratchasima, Thailand; 11 John Innes Centre, Norwich Research Park, Norwich, UK; 12 Department of Chemistry, Faculty of Sciences, Mansoura University, Mansoura, Egypt; 13 Center for Biosustainability, Danish Technical University, Lyngby, Denmark

**Keywords:** Metabolic engineering, Biosynthesis

## Abstract

QS-21 is a potent vaccine adjuvant and remains the only saponin-based adjuvant that has been clinically approved for use in humans^1,2^. However, owing to the complex structure of QS-21, its availability is limited. Today, the supply depends on laborious extraction from the Chilean soapbark tree or on low-yielding total chemical synthesis^3,4^. Here we demonstrate the complete biosynthesis of QS-21 and its precursors, as well as structural derivatives, in engineered yeast strains. The successful biosynthesis in yeast requires fine-tuning of the host’s native pathway fluxes, as well as the functional and balanced expression of 38 heterologous enzymes. The required biosynthetic pathway spans seven enzyme families—a terpene synthase, P450s, nucleotide sugar synthases, glycosyltransferases, a coenzyme A ligase, acyl transferases and polyketide synthases—from six organisms, and mimics in yeast the subcellular compartmentalization of plants from the endoplasmic reticulum membrane to the cytosol. Finally, by taking advantage of the promiscuity of certain pathway enzymes, we produced structural analogues of QS-21 using this biosynthetic platform. This microbial production scheme will allow for the future establishment of a structure–activity relationship, and will thus enable the rational design of potent vaccine adjuvants.

## Abstract (web summary)

QS-21—an FDA-approved vaccine adjuvant—and several structural analogues of QS-21 can be synthesized in engineered yeast strains, and this process is much less laborious compared with the conventional mode of extraction from the Chilean soapbark tree.

## Main

Adjuvants increase the efficacy of vaccines by stimulating or augmenting the human immune response to pathogens or disease-specific antigens^5^. Ever since its discovery in the 1920s, alum (aluminium hydroxide) has been the most widely used, clinically approved vaccine adjuvant^6^. More recently, QS-21 has been shown to exhibit potent immunoactivity^3,4,7^, and it is an active ingredient in the Adjuvant System AS01 and Matrix M (refs. ^1,2^). These formulations have been approved for GSK’s malaria (Mosquirix) and shingles (Shingrix) vaccines, as well as for Novavax’s COVID-19 vaccines. Motivated by the potent immune response and favourable safety profiles, researchers have since tested QS-21 in more than 120 clinical trials.

**Fig. 1:**
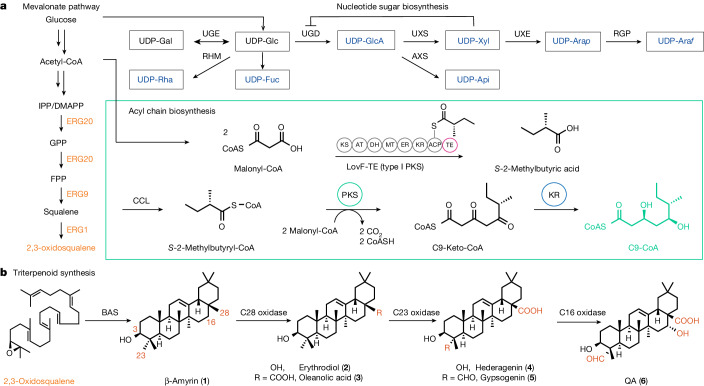
Complete biosynthetic pathway for the de novo production of QS-21 in yeast from simple sugars. Native yeast genes and enzymes that have been overexpressed are shown in orange, and heterologous genes and enzymes are shown in black and navy. **a**, Pathways for the biosynthesis of the QS-21 precursors 2,3-oxidosqualene, UDP-sugars and acyl C9-CoA. DMAPP, dimethylallyl pyrophosphate; GPP, geranyl pyrophosphate; IPP, isopentenyl pyrophosphate; UXE, UDP-xylose epimerase. **b**, Pathways for the synthesis and oxidation of β-amyrin (**1**) to QA (**6**) through six oxidation steps on β-amyrin carried out by three cytochrome P450s. The resulting QA (**6**) is functionalized with C28 carboxylic acid, C23 aldehyde and C16 hydroxy functional groups.

**Fig. 2:**
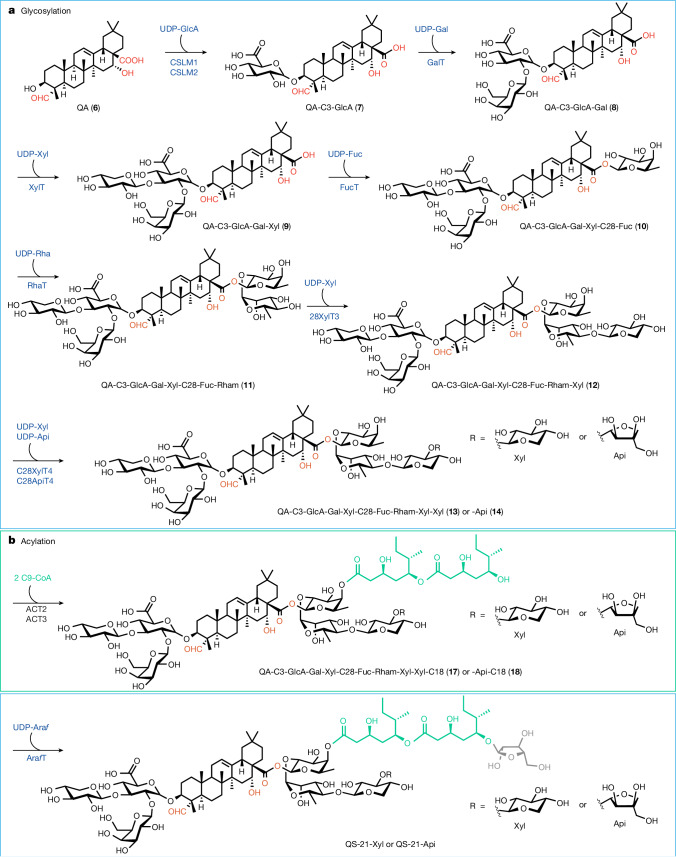
Functionalization of QA (6) to yield QS-21-Xyl and QS-21-Api. **a**, The C3-OH of **6** is decorated with a branched trisaccharide β-d-Xyl-(1→3)-[β-d-Gal-(1→2)]-β-d-GlcA through three sequential glycosylation steps. The C28-COOH is glycosylated with a linear tetrasaccharide β-d-Xyl-(1→4)-α-l-Rha-(1→2)-β-d-Fuc with a terminal sugar of β-d-Xyl or β-d-Api. **b**, The fucose ester-linked to C28 is then acylated twice with a nine-carbon branched dihydroxy acid moiety (C9-CoA), before it is α-l-arabinofuranosylated to complete the biosynthesis of QS-21 in yeast. Information for all genes is listed in Supplementary Table 1. Source Data

Despite major commercial interest, the availability of QS-21 remains limited, owing mainly to its structural complexity^8^. QS-21 consists of four distinct structural domains: (i) a lipophilic triterpenoid core, quillaic acid (QA, Fig. 1), flanked by (ii) a branched trisaccharide moiety on the C3 position, (iii) a linear tetrasaccharide chain on the C28 position and (iv) an unusual pseudodimeric acyl chain capped by an arabinofuranose (Fig. 2). Owing to the structural similarity of two isoforms, QS-21 exists as a heterogeneous mixture of QS-21-Api and QS-21-Xyl in a 65:35 ratio, with the sole difference being the C28 terminal sugar. Traditionally, QS-21 is extracted from the tree bark of the soapbark tree *Quillaja saponaria*, which is native to Chile. Isolation is complicated because the plant extract contains a multitude of different structurally related *Quillaja* saponins, rendering the purification process highly laborious and low yielding^3^. Using an intermediate saponin as the starting material, the total chemical synthesis of both the Xyl and the Api forms of QS-21 has been achieved^4,5^. However, the synthetic route requires 76 steps, and the overall yield is negligible. Thus, developing alternative production processes that are more sustainable and scalable would help to meet the ever-increasing demand for potent vaccine adjuvants, and to address existing or emerging medical needs.

The genes and enzymes for the QS-21 biosynthetic pathway have only been characterized from *Q. saponaria* recently^9,10^. Here we report the complete biosynthesis of QS-21-Api and QS-21-Xyl, as well as their structural derivatives in *Saccharomyces cerevisiae*, starting from only simple sugars (glucose and galactose). To accomplish this, we first upregulated the yeast native mevalonate pathway to provide a high carbon flux towards 2,3-oxidosqualene, which is then cyclized by a heterologous β-amyrin synthase and site-selectively oxidized by plant cytochrome P450s to yield QA, the aglycone of QS-21. We further introduced plant nucleotide sugar synthetic pathways to make seven non-native uridine diphosphate sugars (UDP-sugars), which are used to add sugars onto the C3 hydroxy and C28 carboxy functional groups of QA through the co-expression of QS-21 pathway glycosyltransferases (GTs)^9^. Furthermore, an engineered type I polyketide synthase (PKS), two type III PKSs and two stand-alone ketoreductases (KRs) were expressed in yeast to form the dimeric acyl unit that constitutes the last step before the terminal arabinofuranose addition to yield QS-21 (ref. ^10^) (Fig. 1a and Fig. 2b). Pathway enzymes, as well as their functional homologues from various plants that produce structurally similar saponins (for example, *Saponaria vaccaria*), fungi (LovF from *Aspergillus terreus*) and bacteria, were functionally expressed in the engineered yeast. This combinatorial approach allowed us to select the activities that function optimally together in a yeast cell, thereby enabling the production of QS-21. Owing to the promiscuity of several enzymes, structural analogues of QS-21 were produced using the biosynthetic platform described here; this will enable a structure–bioactivity relationship to be established in the future, and will allow the rational design of potent vaccine adjuvants.

## Biosynthesis of quillaic acid

**Extended Data Fig. 1:**
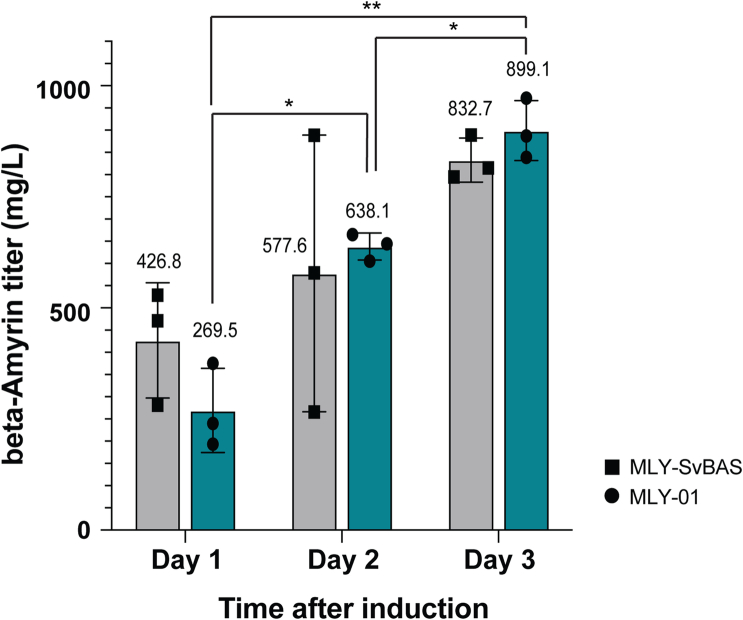
Biosynthesis of β-amyrin in engineered yeast and culture condition optimization. Full-length β-amyrin synthase (BAS) sourced from *Saponaria vaccaria*, *Sv*BAS, was integrated into JWY601, a strain with upregulated mevalonate pathway. Production of β-amyrin was sampled over the course of three days after induction. MLY-01 harbouring overexpressed copies of ERG20 and ERG1 led to 899 mg l^−1^ production of β-amyrin. Data are mean ± s.d.; n = 3 biologically independent samples. *P value < 0.05; ** *P* < 0.01. Student’s two-tailed t-test. More statistical analysis is available in the source data file. Source Data

The *Saccharomyces cerevisiae* strain JWy601 was chosen as the base strain to the triterpene core, β-amyrin, of QS-21. The mevalonate-based isoprenoid biosynthetic pathway in this strain had previously been upregulated to produce sesquiterpenes^11^. In JWy601, all genes encoding enzymes that convert acetyl-CoA to farnesyl pyrophosphate (FPP) were placed under the control of galactose-inducible promoters for controlled overexpression. The culture was grown at first in a glucose-containing rich medium, YPD, for 48 h to maximize the cell mass before a 72-h production phase was initiated by the addition of galactose. β-Amyrin synthases (BASs) of various plant origins (*Artemisia annua*, *Arabidopsis thaliana*, *Glycyrrhiza glabra* and *Saponaria vaccaria*) were integrated into JWY601 to quantify the production of β-amyrin from squalene by gas chromatography–mass spectrometry (GC–MS). Among these candidates, the BAS homologue from *S. vaccaria* (*Sv*BAS) yielded the highest titre of β-amyrin (**1**), the production of which was further confirmed by efficient consumption of squalene compared to the parent strain, JWy601 (Extended Data Fig. 1a,b). Further upregulation of mevalonate pathway genes encoding ERG20 and ERG1 (Fig. 1a) ultimately resulted in a β-amyrin titre of 899.0 mg l^−1^ over a production period of three days (Extended Data Fig. 1c).

**Fig. 3:**
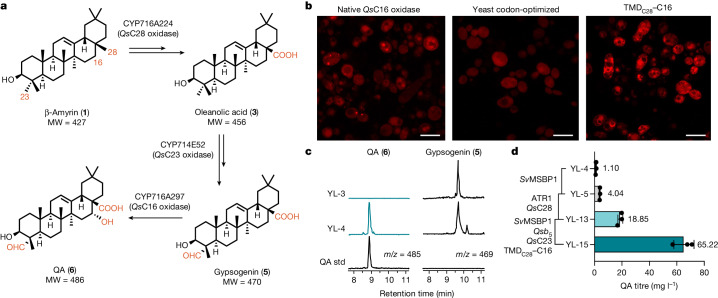
Functional expression of cytochrome P450s and pathway engineering for QA. **a**, Three cytochrome P450s oxidize β-amyrin (**1**) at the C28, C23 and C16 positions, resulting in a carboxylic acid, an aldehyde and a hydroxy group, respectively, on QA (**6**). MW, molecular weight. **b**, When expressed in yeast, the native sequence of C16 oxidase (CYP716A297) encodes a protein that has both soluble and aggregated forms, whereas the C16 oxidase expressed from the yeast codon-optimized sequence is cytosolic. By fusing the TMD of C28 oxidase to the N terminus of C16, the TMD_C28_–C16 fusion protein was correctly anchored to the ER membrane. Images were acquired using a Zeiss LSM 710 confocal microscope. At least three independent experiments were performed. Scale bars, 10 μm. **c**, Functional expression of TMD_C28_–C16 leads to the conversion of gypsogenin (**5**) to QA (**6**). **d**, Metabolic engineering strategies, including the expression of a MBSP, as well as the overexpression of the cytochrome P450s and their redox partners, improved the titre of QA by 60-fold. Data are mean ± s.d.; *n* = 3 biologically independent samples. Source Data

Expression cassettes containing cytochrome P450s identified in *Q. saponaria*^9^ as well as the redox partner, cytochrome P450 reductase (CPR, *At*ATR1 from *A. thaliana*), were integrated sequentially into the yeast genome to achieve the biosynthesis of the triterpenoid core, QA (Fig. 3a). Extraction of the culture medium and analysis by liquid chromatography–mass spectrometry (LC–MS) showed that the CPR was sufficient as a redox partner to facilitate the three-step oxidation at the C28 position to the carboxylic acid carried out by CYP716A224, reaching a titre of 263.4 mg l^−1^ of oleanolic acid (**3**) by strain YL-1 (Extended Data Fig. 3c). By contrast, C23 oxidation required a *Quillaja* native cytochrome *b*_5_ (*Qsb*_5_) reductase for the hydroxy functional group to be oxidized to an aldehyde to yield gypsogenin (**5**, strain YL-3; Supplementary Fig. 1). Cytochromes *b*_5_ have long been known to increase the activities of cytochrome P450 through (i) direct electron transfer from NADH-cytb_5_ to P450s in a pathway independent of NADPH-CPR and (ii) potentially faster transfer of the second electron as compared with CPR^12,13^. Indeed, co-expression of cytochrome *b*_5_, cytochrome P450s and CPRs has yielded higher oxidation efficiencies, leading to higher titres of the oxidized products in heterologous hosts^14–16^. For the C16 oxidation, despite a predicted transmembrane domain at the N terminus of the C16 oxidase, subcellular localization studies revealed that the yeast codon-optimized CYP716A297 (C-terminal mCherry fusion) was cytosolic, and no oxidized product was detected. A different localization pattern with more protein aggregation was observed for the same protein expressed from a gene with the native plant sequence, possibly owing to differences in yeast and plant codon frequencies and concomitant changes in protein translation efficiency (Fig. 3b). To localize P450 to the endoplasmic reticulum (ER) membrane, the predicted 22-amino-acid transmembrane domain (TMD) of the C28 oxidase was fused to the N terminus of the C16 oxidase, thereby creating the fusion protein TMD_C28_–C16 and resulting in the production of QA (**6**) at 1.1 mg l^−1^ (strain YL-4) (Fig. 3c).

**Extended Data Fig. 2:**
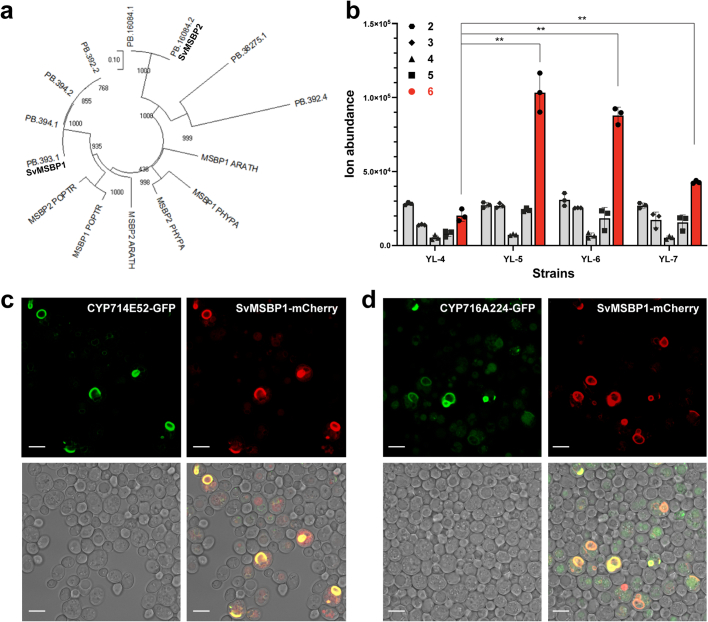
Optimization of P450 oxidation efficiency through the expression of a scaffolding MSBP. **a**, Phylogenetic tree of *Saponaria vaccaria* MSBP homologues and related MSBPs (see details in the Supplementary Methods). **b**, Expression of various MSBP homologues, including 2 newly identified *Sv*MSBPs, led to 2- to 4-fold increase of QA production. Increased of QA production can be attributed to the improved oxidation efficiency. Data are mean ± s.d.; n = 3 biologically independent samples. *P value < 0.05; ** *P* < 0.01. Student’s two-tailed t-test. More statistical analysis is available in the source data file. Subcellular localization studies show that *Sv*MSBP1 co-localizes with both **c**, C28 and **d**, C23 oxidases on the ER membrane of yeast. Images were acquired using a Zeiss LSM 710 confocal microscope (scale bar represents 10 μm; at least three independent experiments were conducted). Source Data

**Extended Data Fig. 3:**
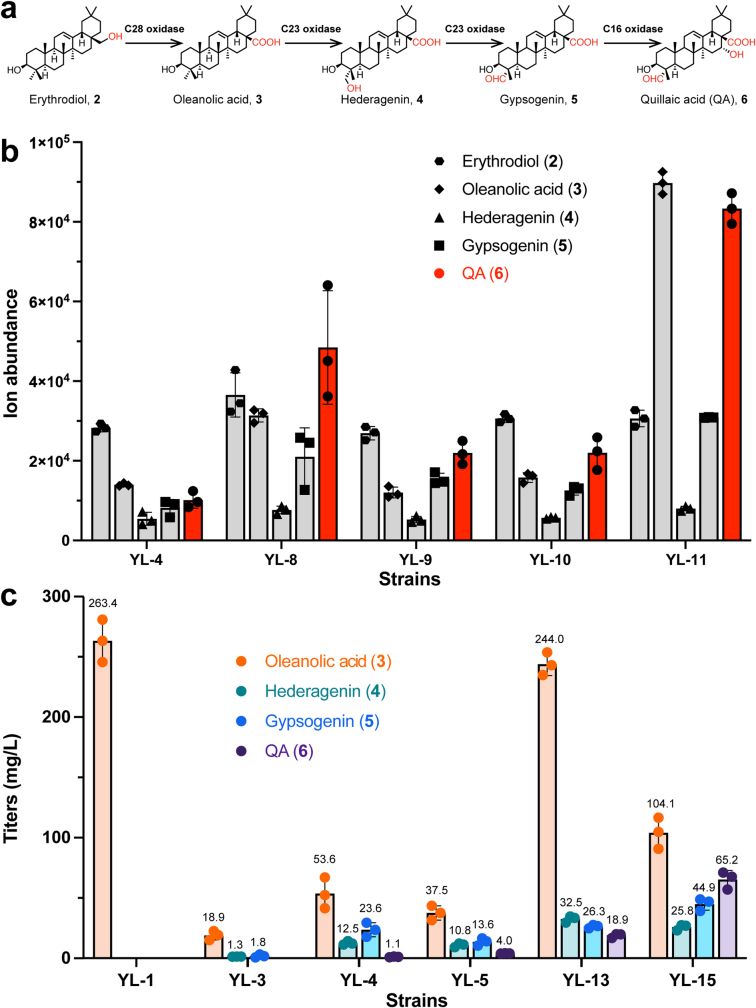
Functional expression of cytochrome P450s and pathway engineering for QA. **a**, Introduction of additional copies of pathway enzymes revealed reaction bottlenecks and the increased production of QA. **b**, Distribution of the terpenoid products in the engineered yeast strains after overexpression of the cytochrome P450s, as well as their redox partners. **c**, Calculated titres of target products in these yeast strains with a 65-fold increase of QA production in YL-15 compared to YL-4. Data are mean ± s.d.; n = 3 biologically independent samples. Source Data

To optimize the P450 oxidation efficiency, we opted for the introduction of a membrane steroid-binding protein (MSBP) to act as a scaffold for co-localization of the P450s. Despite their spatial proximity on the ER membrane, cytochrome P450s do not directly interact with each other. In plants, MSBPs serve an important physiological role in regulating lignin biosynthesis in *A. thaliana* by establishing physical interaction with and organizing the pathway P450s^17^. Indeed, production of the final oxidation product QA increased by fourfold after the expression of a newly identified MSBP candidate from *S. vaccaria* (Extended Data Fig. 2a,b, Supplementary Methods, Supplementary Fig. 2 and Supplementary Table 5). Subcellular localization studies revealed that *Sv*MSBP1 co-localizes with both C28 and C23 oxidases on the ER membrane (Extended Data Fig. 2c,d). Such spatial proximity further corroborates its scaffolding function for non-lignin-related P450s and thus constitutes an efficient and potentially universal strategy to improve the activities of P450 in heterologous hosts. To identify the bottleneck among the six oxidation steps with three P450 monooxygenases, C28, C23 and TMD_C28_–C16 oxidases were overexpressed individually in a strain that contained a single copy of each P450 and ATR1 integrated into the chromosome (YL-8 to YL-10), leading to a fourfold, twofold and twofold increase, respectively, in QA production (Extended Data Fig. 3). In addition, overexpressing a second copy of the CPR in the C28-overexpressing strain (YL-11) increased the titre of **6** by eightfold, suggesting that the activities of all three P450s and their redox partners are suboptimal. To optimize the production of **6**, two copies of the P450s, redox partners and MSBP were integrated into the strain YL-15 to yield 65.2 mg l^−1^ of **6** in shake flask cultures (Fig. 3d and Extended Data Fig. 3).

## C3 and C28 *O*-glycosylation

The final product QS-21 is a water-soluble triterpene glycoside with an amphiphilic character—a prerequisite for homogenous mixtures with soluble antigen in the vaccine formulation^18^. It is the sugar decorations on the C3 hydroxy and C28 carboxylic acid groups that render the non-polar triterpene core **6** hydrophilic. The complete glycosylation of QS-21 requires eight glycosylating steps, involving seven different UDP-sugars (that is, UDP-d-glucuronic acid (UDP-GlcA), UDP-d-galactose (UDP-Gal), UDP-d-xylose (UDP-Xyl), UDP-d-fucose (UDP-Fuc), UDP-l-rhamnose (UDP-Rha), UDP-d-apiose (UDP-Api) and UDP-l-arabinofuranose (UDP-Ara*f*)). Among these, UDP-Gal is the only UDP-sugar that is native to yeast and can be obtained through galactose metabolism or UDP-glucose isomerization (Fig. 1a). As such, heterologous nucleotide sugar synthases were introduced into the yeast host^19^ along with their corresponding GTs in a stepwise manner. The detection of each glycosylated product confirmed the functional expression of both the sugar synthases and the transferases.

**Extended Data Fig. 4:**
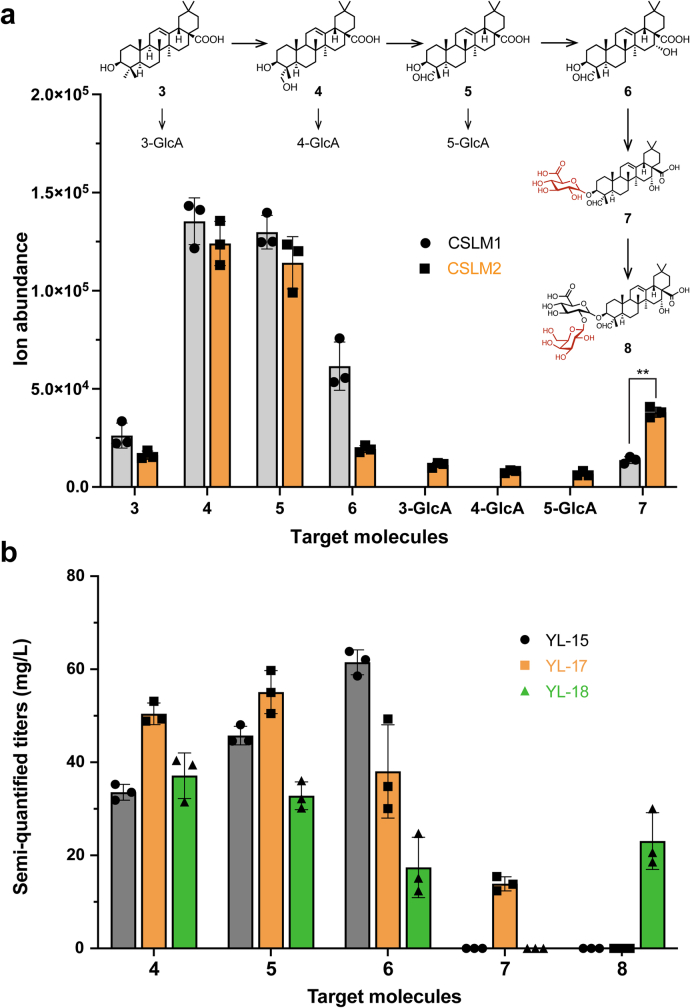
C3 glycosylation studies. **a**, Activity of the two CSL homologues CSLM1 and CSLM2 was investigated by comparing the LC–MS ion intensity of the oxidized intermediates oleanolic acid (**3**), hederagenin (**4**), gypsogenin (**5**), QA (**6**), as well as their glucuronidated counterparts in the presence of CSLM1 or CSLM2 (shown in grey and orange, respectively). CSLM1 was more specific towards the glucuronidation of **6**. Although CSLM2 can also glucuronidate less oxidized intermediates such as **3**, **4**, and **5**, it was 3-fold more active towards **6**. *P value < 0.05; ** *P* < 0.01. Student’s two-tailed t-test. More statistical analysis is available in the source data file. **b**, Distribution of the glycosylated products and their precursors in the engineered yeast strains. Expression of the downstream C3-GalT effectively increased the conversion of **6** by pushing the equilibrium through the consumption of **7**, thus leading to the production of **8**. Data are mean ± s.d.; n = 3 biologically independent samples. Source Data

**Extended Data Fig. 5:**
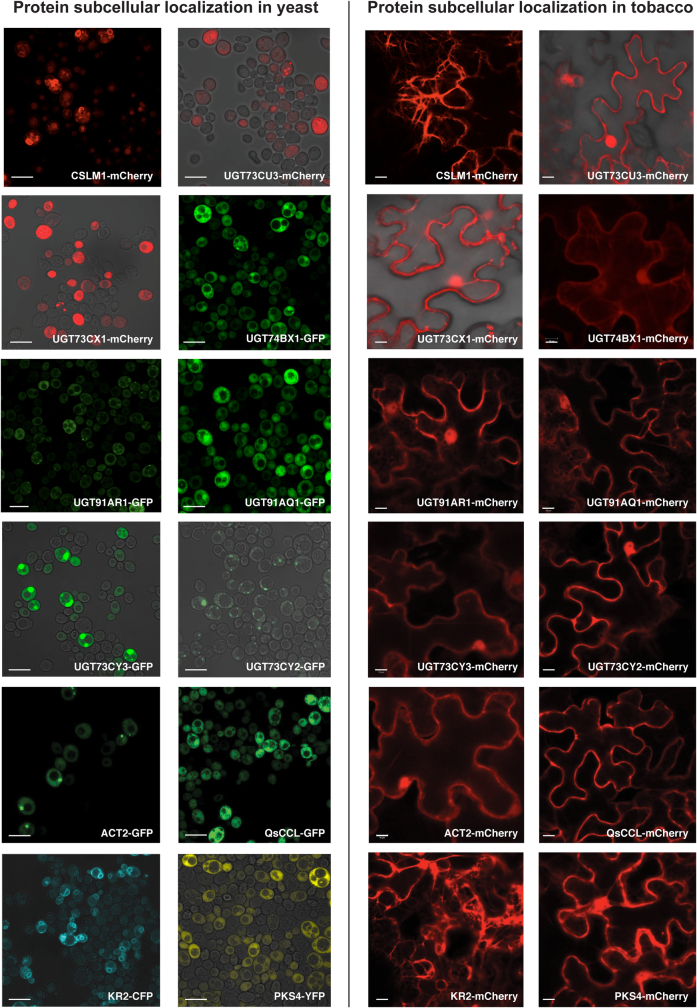
Subcellular localization studies of QS-21 pathway proteins in yeast and tobacco. C-terminal fluorescent protein fusions of the pathway enzymes are visualized. The first GT CSLM1 was localized in the ER membrane and the downstream enzymes were expressed in the cytosol except for KR2. Images were acquired using a Zeiss LSM 710 confocal microscope (scale bar represents 10 μm).

Two *Q. saponaria* GTs belonging to the cellulose synthase-like family of enzymes have been identified that add glucuronic acid to QA to give 3-*O*-{β-d-glycopyranosiduronic acid}-QA (CSLM1 and CSLM2)^9^. After co-expression of a UDP-glucose dehydrogenase from *A. thaliana* (*At*UGD1) with CSLM1 (YL-16) or CSLM2 (YL-17), a new LC–MS peak that corresponds to the exact mass of **7** was detected. We observed that CSLM1 is more specific to the glucuronidation of **6**, whereas CSLM2 can also glucuronidate less oxidized intermediates such as **3**, **4** and **5**, but is threefold more active towards **6** (Extended Data Fig. 4a and Supplementary Fig. 3). Therefore, CSLM2 was chosen for further pathway engineering. The first glycosylation step takes place in the ER membrane, along with the previous oxidation steps and the formation of the triterpenoid substrates (Extended Data Figs. 2 and 4). Both CSLMs were predicted to have seven transmembrane domains and confocal microscopy studies in both yeast and tobacco further confirmed the localization in the ER. It was also observed, when preparing the standards, that the glucuronidation of **6** substantially increases its water solubility. We speculate that **7** migrates to the cytoplasm, where the GTs are localized, to carry out the subsequent six C3 and C28 glycosylation steps (Extended Data Fig. 5).

**Fig. 4:**
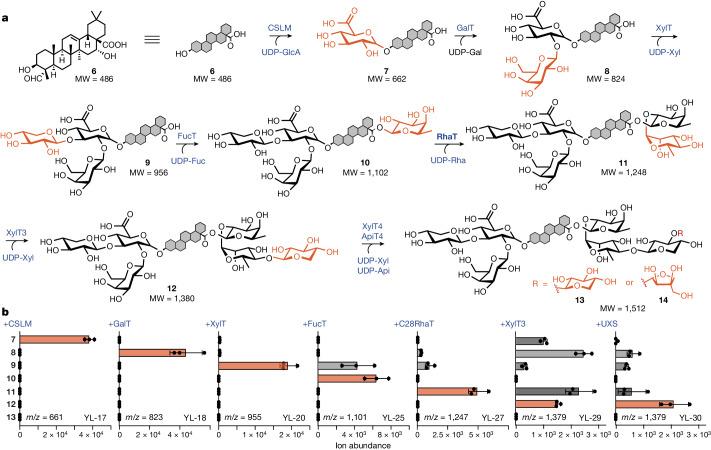
Reconstitution of the glycosylation pathway by the functional expression of nucleotide sugar synthases and corresponding GTs. **a**, Sequential addition of the C3 branched trisaccharide (GlcA-Gal-Xyl) before a linear tetrasaccharide is added stepwise to the C28 carboxylic acid (Fuc-Rha-Xyl-Xyl or Fuc-Rha-Xyl-Api). **b**, LC–MS peak area of corresponding products produced in yeast after the expression of the indicated enzymes and the necessary nucleotide sugar synthases. The bars in red and grey indicate the ion abundance of the target molecules and intermediates, respectively. Data are mean ± s.d.; *n* = 3 biologically independent samples.

The second glycosylation step of the C3 position is carried out by the cytosolic enzyme UGT73CU3 (C3-GalT), which efficiently galactosylates **7** by 1,2-glycosidic bond formation to yield **8** (Fig. 4, Extended Data Fig. 4b and Supplementary Fig. 4). When expressed alone, CSLMs cannot exhaust the pool of **6**. However, expression of the downstream C3-GalT increased the conversion of **6** by pushing the equilibrium through the consumption of **7**, thus leading to the production of **8** at 24.3 mg l^−1^ (strain YL-18; Extended Data Fig. 4b). When UDP-xylose synthase (*At*UXS3) was expressed in conjunction with UGT73CX1 (XylT) and the unmodified *At*UGD1, no glycosylated product (that is, **7**, **8** or **9**) was observed (Supplementary Fig. 5a). This can be rationalized by the common feedback mechanism in which UDP-Xyl strongly inhibits UGDs to maintain the homeostasis of the intracellular UDP-Glc pool^20,21^. To alleviate this inhibitory effect, an A101L mutation^22^ was introduced into *At*UGD1 (strain YL-20) to reduce feedback inhibition by UDP-Xyl, thus allowing the xylosylation of **8** to yield **9** (Fig. 4 and Supplementary Fig. 5b).

The C28 linear tetrasaccharide assembly follows a sequential order of d-fucose, l-rhamnose and d-xylose, as well as d-xylose or d-apiose as the terminal sugar. The d-fucose is linked to the C28 carboxylic acid functional group of QA by an esterification facilitated by a GT belonging to the GT1 family (UGT74BX1, C28FucT), with a UDP-sugar as the substrate. The biosynthetic pathway of UDP-d-Fuc, in which UDP-glucose is converted to UDP-4-dehydro-6-deoxy-d-glucose through UDP-glucose 4,6-dehydratase (*Sv*UG46DH), has been reported only recently^23^. C28FucT adds the UDP-deoxy-sugar, which is then reduced once it has been added onto the **9** backbone by FucSyn^9^, leading to the C28-fucosylated product **10** after the expression of all three enzymes (*Sv*UG46DH, FucSyn and C28FucT in strain YL-25). Although residual **9** was observed in the presence of UGT74BX1 alone, the expression of a UDP-l-rhamnose synthase (*At*RHM2) and the downstream UGT91AR1 (C28RhaT) helped to fully convert **10** to **11** (strain YL-27), thus efficiently pulling the equilibrium of C28FucT and increasing pathway flux (Fig. 4b). Pathway intermediates, in particular **8** and **11**, started to accumulate after the expression of UGT91AQ1 (C28XylT3, strain YL-29). The fact that they are substrates for two xylosyltransferases (C3XylT and C28XylT3) indicated that UDP-Xyl was the limiting factor (Fig. 4b and Supplementary Fig. 6). As such, an additional copy of *At*UXS was integrated and expressed (strain YL-30), which effectively relieved the accumulation of C3-glycosylated products and enabled the production of **12** as the major product. The last glycosylation step on the C28 position suffers from the tendency of both UGT73CY3 (C28XylT4) and UGT73CY2 (C28ApiT4) to misfold in yeast, leading to only trace amounts of the fully glycosylated products **13** and **14** in strains YL-33 and YL-34, respectively (Supplementary Fig. 7). These two enzymes have high protein sequence homology (94.89%) and thus, might display similar stability when expressed in a heterologous host. Subcellular localization studies of C-terminally GFP-tagged UGT73CY2 and UGT73CY3 revealed that although they are correctly localized to the cytoplasm at early stages of expression, aggregated forms adjacent to the vacuole become the dominant species with culture time (Supplementary Fig. 8c). However, when fresh carbon and nitrogen resources are provided (that is, fresh YP galactose), the expression of protein under galactose-inducible promoters is switched on when an additional inducer (galactose) is added to the medium, leading to higher cytosolic expression of UGT73CY3.

## Biosynthesis and addition of the acyl unit

**Fig. 5:**
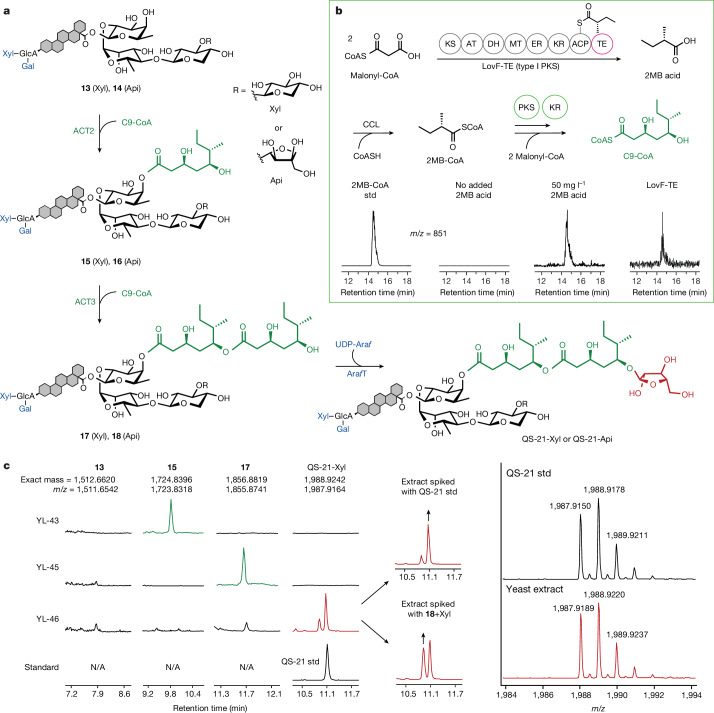
Acylation and terminal glycosylation towards the complete biosynthesis of QS-21. **a**, The C9-CoA unit is synthesized by converting 2MB-CoA and two equivalents of malonyl-CoA through the functional expression of type III PKSs and two KRs. 2MB-CoA is not native to yeast but can be obtained by activating 2MB acid through CoA thioesterification, which can be supplemented directly in the culture medium or biosynthesized through an engineered type I PKS LovF-TE. **b**, Biosynthetic pathway of C9 acylation to the glycosylated **13** and **14** to form a repeating dimeric C18 moiety before the terminal arabinofuranose is added to the 5-OH. **c**, Ion-extracted chromatograms of the extracted yeast samples in which the engineered strains were grown in the presence of 2MB showed the efficient addition of both C9 units onto the glycosylated molecule substrate (**13**) to yield acylated **13**-C9 (**15**), and **13**-C18 (**17**). The arabinofuranosylation of **17** led to the biosynthesis of QS-21-Xyl, which co-elutes with the QS-21 chemical standard. The identical isotopic fingerprint patterns further confirm the in vivo production of QS-21. The extracted peak preceding that of QS-21 corresponds to **18**+Xyl generated in vitro, possibly owing to the promiscuity of the Ara*f* transferase.

The specific immunological role of the acyl group in QS-21 remains unclear, but structure–activity relationship studies have shown that it is crucial to the potent activity of QS-21 in stimulating and soliciting cytokine responses mediated by T helper 1 cells^24,25^. The biosynthesis of each of the dimeric C9 acyl chains requires two consecutive decarboxylative Claisen condensation reactions of malonyl-CoA with (*S*)-2-methylbutyryl-CoA (2MB-CoA; Fig. 5). This is catalysed by two type III polyketide synthases, PKS4 and PKS5, with the keto intermediate being reduced by two stand-alone ketoreductases, KR1 and KR2, to form the 3,5-dihydroxy moiety in C9-CoA (ref. ^10^) (Fig. 1b). No native metabolic pathway in yeast involves 2MB-CoA, and free 2MB acid was therefore first added exogenously to the culture medium at 50 mg l^−1^, with the heterologous expression of a *Quillaja* short chain fatty acid CoA ligase (*Qs*CCL1), to yield 2MB-CoA in YL-*Qs*CCL (Fig. 5b).

**Extended Data Fig. 6:**
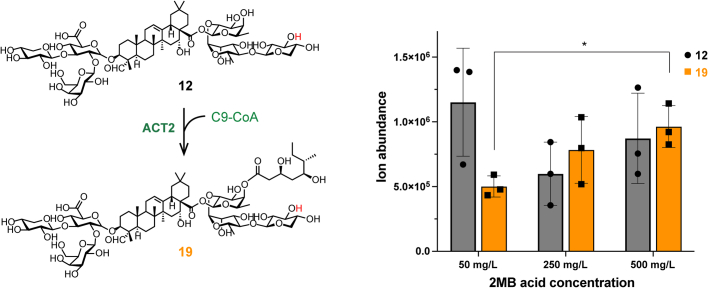
The C9 acylation of 12 in the presence of exogenously supplemented 2MB acid. The conversion of **12** to **19** was limited possibly due to the limited availability of intracellular 2MB acid. Increased concentration of 2MB added to the culture medium effectively improved the conversion and the concentration of 2MB supplementation is thus determined to be 500 mg l^−1^. Data are mean ± s.d.; n = 3 biologically independent samples. *P value < 0.05. Student’s two-tailed *t*-test. More statistical analysis is available in the source data file. Source Data

**Extended Data Fig. 7:**
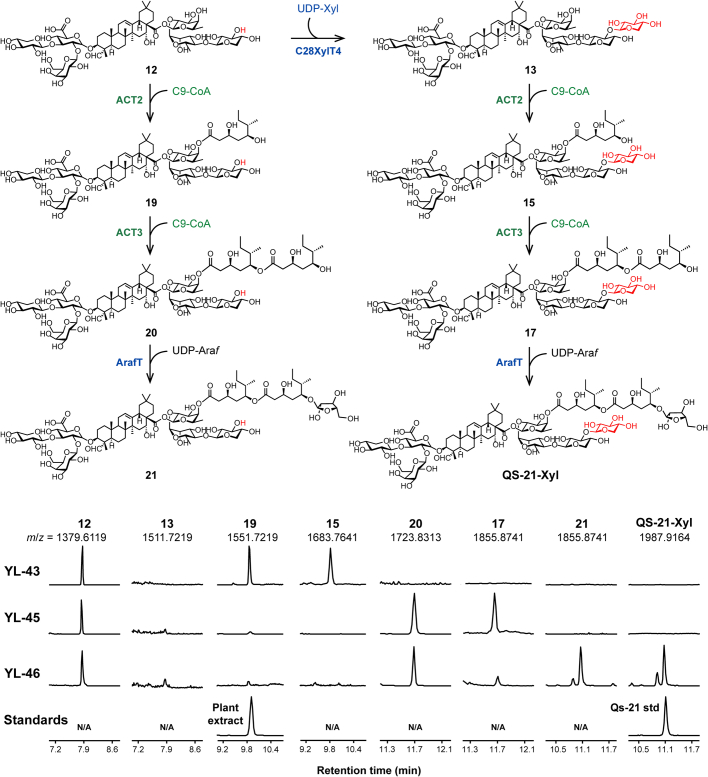
Acylated and glycosylated intermediates towards the complete biosynthesis of QS-21. The C9-CoA unit can be added to both the C28 trisaccharide **12** and tetrasaccharide **13** with the same acyl transferase ACT2. ACT3, which catalyses the second acylation to form a repeating dimeric C18 structural motif, used both **19** and **15** as substrates, before the terminal arabinofuranose was added to the 5-OH of the C18 group. Bottom, ion-extracted chromatograms of the extracted yeast samples showed the efficient addition of the two C9 units onto the glycosylated molecule substrates (**12** and **13**) to yield fully acylated **12**-C18 (**20**), and **13**-C18 (**17**). The arabinofuranosylation of **20** and **17** led to the biosynthesis of **21** and QS-21-Xyl. **21**, a structural derivative of QS-21 accumulated in yeast due to the competitive pathways between glycosylation and acylation.

The acyl biosynthetic cassette (PKS4, PKS5, KR1 and KR2) was first tested in YL-*Qs*CCL, which can make 2MB-CoA intracellularly, but no production of C9-CoA could be detected directly by LC–MS, owing possibly to its chemical instability and potential cyclization into the lactone. Although ACT2 has been reported to efficiently convert the hepta-glycosylated **13** to **15** (Fig. 5a), it is also active on the hexa-glycosylated **12** (ref. ^10^) (Fig. 1). Therefore, the acyl biosynthetic cassette and the first acyl transferase ACT2 were first integrated into the **12**-producing yeast strain (YL-30). In the presence of 2MB acid supplementation to the culture medium, the mono-acylated product **19** was detected by LC–MS, which was confirmed by its co-elution with a tobacco extract standard^10^ (strain YL-42; Extended Data Figs. 6 and 7). Because residual substrate **12** was still detected after the acylation, an increased concentration of 2MB acid was added to the culture medium up to 500 mg l^−1^, which resulted in a much-improved acylation conversion (Extended Data Fig. 6). After the expression of C28XylT4 (strain YL-43), although the mono-acylated hepta-glycosylated product **15** was observed using the culture scheme developed above, residual **12** and **19** were still present in the medium extract, indicating that the terminal xylosylation still requires improvement (Extended Data Fig. 7). The second acyl transferase ACT3 fully acylates both mono-acylated products (**15** and **19**) with an additional C9 unit, resulting in **17** and **20**, respectively (strain YL-45). This indicates that the MB acid supplement and the yeast endogenous malonyl-CoA pool provide sufficient C9-CoA for the two-step acyl biosynthesis and additions.

**Extended Data Fig. 8:**
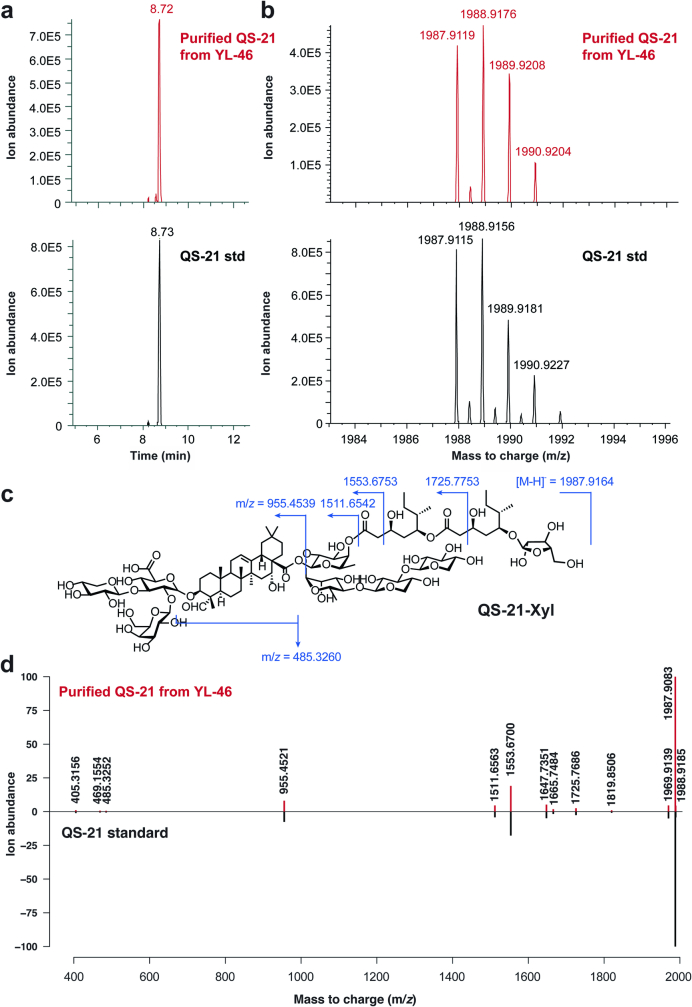
Characterization of purified QS-21-Xyl from engineered yeast YL-46 compared to that of the QS-21 standard. QS-21-Xyl production was scaled up according to the protocol in the Supplementary Information to afford purified QS-21-Xyl. **a**, EIC spectra of QS-21-Xyl extracted from YL-46 cultures show the co-elution with the standard. **b**, Isotopic fingerprint patterns of the QS-21-Xyl extracted from YL-46 cultures and purified (top) and the QS-21 standard (bottom). **c**, Structure of QS-21-Xyl with the characteristic mass fragments are indicated. **d**, Mirror plot comparison of MS2 spectra of QS-21 extracted from YL-46 cultures (top) and of the standard (bottom). A list of the MS2 fragments and their corresponding intensities can be found in Supplementary Table 7. The fragments observed are consistent with those reported in literature^10^. All LC–MS chromatograms were extracted with the theoretical m/z values of the respective compounds of interest.

**Extended Data Fig. 9:**
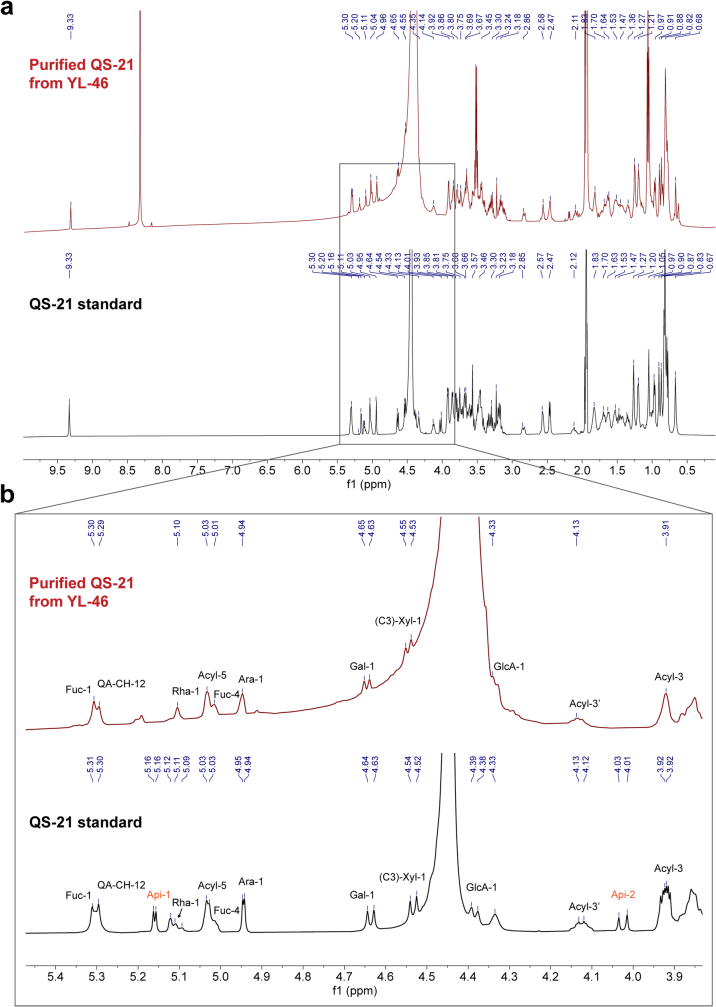
^1^H NMR spectra of purified QS-21-Xyl from engineered yeast YL-46 compared to that of the QS-21 standard. **a**, Full spectral comparison between the QS-21 extracted and purified from engineered yeast YL-46 (top, red) and a QS-21 standard (bottom, black) shows an overall match of all proton peaks. In particular, the peak at chemical shift 9.33 demonstrates the presence of the C23 aldehyde in the QS-21 made in engineered yeast. **b**, Expanded spectral comparison between the purified QS-21 from YL-46 (top, red) and a QS-21 standard (bottom, black). The well-matched anomeric proton peaks confirm the correct connectivity between the sugar moieties. The absence of apiose proton peaks, highlighted in orange, in the yeast sample further confirms the production of QS-21-Xyl exclusively, owing to the specificity of the GT. Both spectra were recorded in acetonitrile-*d*_3_:D_2_O 1:1, 500 MHz.

Plant UDP-l-Ara*f* biosynthesis is closely associated with the Golgi apparatus because l-Ara*f* is a key component in the plant cell wall^26^. The biosynthesis of UDP-arabinopyranose (UDP-Ara*p*) occurs mainly through the epimerization of UDP-Xyl in the Golgi lumen; UDP-Ara*p* is then interconverted into UDP-Ara*f* by UDP-Ara mutase, which is located outside on the cytosolic surface of the Golgi. The resultant UDP-Ara*f* is transported back to the Golgi lumen for its later glycosylation applications^27^. Owing to the lack of yeast native sugar transporters in the Golgi membrane, cytosolic homologues of these nucleotide sugar synthases were chosen. UDP-glucose epimerase 1 from *A. thaliana* (*At*UGE1), a bifunctional enzyme that epimerizes UDP-Glc and UDP-Gal, as well as UDP-Xyl and UDP-Ara*p*, and reversibly glycosylated polypeptide 1 (*At*RGP1), which converts UDP-Ara*p* to UDP-Ara*f*, were expressed to produce UDP-Ara*f* in vivo. Integration and expression in the presence of UGT73CZ2 (Ara*f*T) led to a new LC–MS peak that corresponds to the exact mass of 1987.9164 and co-elutes with the QS-21 standard (Fig. 5c); this was further corroborated and confirmed by the identical isotopic fingerprint patterns of the extracted sample and QS-21. Note that two mass peaks were observed in the extracted LC–MS spectrum. When spiked with QS-21 standard, or **18**+Xyl (QS-21 with an acyl terminal Xyl instead of Ara*f*) individually^10^, the two LC–MS peaks were successfully identified as Xyl- or Ara*f*-capped **17**, respectively, with the latter being QS-21-Xyl (Fig. 5c) produced at 94.6 ± 8.3 μg l^−1^ in YL-46. To further confirm the biosynthesis of QS-21-Xyl in YL-46, the production was scaled up to allow sufficient materials to be purified and characterized by high-resolution tandem mass spectrometry (HRMS2; Supplementary Methods and Supplementary Table 6) and ^1^H nuclear magnetic resonance (NMR). The identical fragments observed in the MS2 spectra of purified QS-21-Xyl from YL-46 and in those of the standard, along with the mirroring corresponding ion intensities, provide evidence that they have the same structural composition (Extended Data Fig. 8 and Supplementary Table 7). In addition, the well-matched overall spectrum and, in particular, anomeric proton peaks confirm the correct structure and connectivity between sugar moieties (Extended Data Fig. 9). A similar strategy was used to engineer YL-47 to produce QS-21-Api—the same gene cassettes of C9-CoA and UDP-Ara*f* biosynthesis and addition were integrated into the **14**-producing strain to yield QS-21-Api at 31.1 ± 0.5 μg l^−1^ in YL-47 (Supplementary Fig. 9).

To realize the complete biosynthesis of QS-21 from the simple sugar galactose without exogenous supplementation of 2MB acid, we first sought to express the branched-chain α-keto acid dehydrogenase complex with a transaminase from *Bacillus subtilis*, which readily converts isoleucine to 2MB-CoA during amino acid metabolism. However, no 2MB-CoA was detected in yeast, which is probably due to the fact that yeast lacks the necessary post-translational modification mechanism of subunit E2 of the cluster^28^. Alternatively, a 7.6-kb gene encoding the type I PKS protein F (LovF) from the Lovastatin biosynthesis cluster from *Aspergillus terreus* was used to source 2MB-CoA intracellularly. This megasynthase converts two units of malonyl-CoA to 2MB covalently attached to the acyl carrier protein (ACP) domain, which would be directly transferred onto the lovastatin acid precursor monacolin in the native lovastatin pathway^29^. We engineered LovF by truncating it after ACP and fusing it to the promiscuous erythromycin PKS (EryPKS) M6 thioesterase (TE)^30^ through an interdomain linker. As a result, methylbutyryl-S-ACP was hydrolysed to release free 2MB. In the yeast that contained a chromosomal copy of the phosphopantetheinyl transferase (*npgA*)^29^, detectable amounts of 2MB-CoA were observed in the LC–MS traces when LovF-TE and *Qs*CCL were co-expressed (Fig. 5b), thus demonstrating the successful engineering of a PKS that catalyses the release of free 2MB acid from the LovF ACP domain and its subsequent CoA activation. The 2MB-CoA cassette was integrated into YL-46 and YL-47, leading to the production of QS-21-Xyl and QS-21-Api biosynthesized from only simple sugars (strains YL-50 and YL-51; Supplementary Fig. 10).

## Discussion

In addition to the upregulated yeast native mevalonate pathway, our final strain contains 38 heterologous enzymes sourced from six species, spanning several enzyme families: a terpene synthase, P450s, nucleotide sugar synthases, GTs and acyl transferases, as well as type I and type III PKSs. To achieve the complete biosynthesis of QS-21, we mimicked in yeast the subcellular compartmentalization of plants from the ER membrane to the cytosol. QS-21-Xyl and QS-21-Api—two isomers of QS-21 with high structural similarity—can therefore be produced in separate yeast strains, and this enables them to be purified, and their immunoactivity to be characterized, in an independent manner.

**Extended Data Fig. 10:**
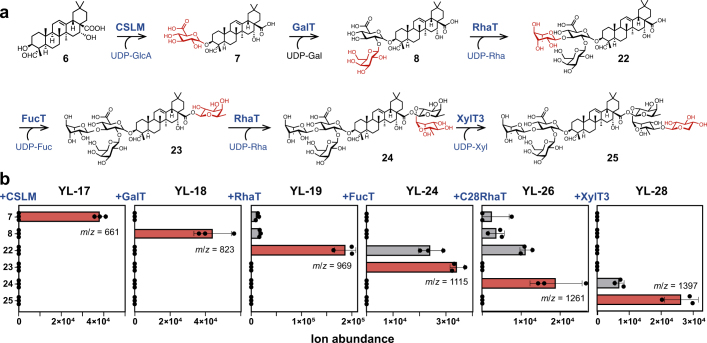
Reconstitution of the glycosylation pathway of QS-21 with a C3 terminal rhamnose structural analogue. **a**, The sequential addition of the C3 branched trisaccharide (GlcA-Gal-Rha) before a linear tetrasaccharide is added stepwise to the C28 carboxylic acid (Fuc-Rha-Xyl) by the functional expression of nucleotide sugar synthases and corresponding GT. The C28 GTs are promiscuous and can also use the Rha trisaccharide derivative. **b**, The LC–MS peak area of corresponding products produced in yeast after the expression of the indicated enzymes and the necessary nucleotide sugar synthases. Data are mean ± s.d.; n = 3 biologically independent samples. Source Data

Moreover, the yeast biosynthetic platform provides vast opportunities to produce structural variants of QS-21 by expressing alternative pathway enzymes or by making fragments of QS-21, exploiting the promiscuity of the enzyme in the pursuit of new leads for vaccine adjuvants. For example, the xylose in the C3 trisaccharide cluster can be replaced by rhamnose, with an additional methyl group, by expressing a rhamnose transferase (UGT73CX2, C3RhaT) instead of the xylose transferase described above (Extended Data Fig. 10). The rhamnose-containing trisaccharide **22** is also a substrate for downstream pathway enzymes and can easily yield a methylated QS-21 derivative. When investigating the glycan functions of QS-21, GTs can be intentionally left out to yield truncated oligosaccharides, highlighted here by the successful biosynthesis of **21** (Extended Data Fig. 7).

The traditional method of extraction and purification of QS-21 from the soapbark tree destroys the bark of the tree, and has prompted increased governmental regulations around its deforestation. Our demonstration of the total biosynthesis of QS-21 in an engineered yeast strain highlights the possibility of replacing the plantation-based supply of saponins with industrial fermentation at scale, which could markedly increase the availability of QS-21 to meet the rising demand for potent vaccine adjuvants. At present, strain YL-46 produces approximately 0.0012% w/w QS-21 per dry cell weight, which is less than the w/w yield from the tree (0.0032%; Supplementary Methods), but it does so over a period of days. As a result, the production of QS-21 in yeast is still considerably faster (by approximately 1,000 times) than it is in the native *Q. saponaria*, which produces QS-21 in trees only once they reach an age of 30–50 years^31^. Although key developments in strain engineering, production and fermentation schemes, as well as in the downstream extraction and purification processes, will still be necessary to produce yeast-derived QS-21 at scale, landmark successes in this arena, such as the industrial-scale production of the anti-malarial precursor artemisinic acid^14^, have paved the way for new opportunities in microbial biomanufacturing.

## Methods

### Chemicals

Numbers, trivial names and International Union of Pure and Applied Chemistry (IUPAC) names, as well as chemical structures of pathway metabolites, are listed in Supplementary Table 3. All chemical standards used in this study are analytical grade and are listed in Supplementary Table 4.

### Plasmid construction

All plasmids were constructed by Gibson assembly (New England Biolabs, HiFi DNA Assembly Master Mix), followed by heat shock transformation into *Escherichia coli* DH5α competent cells, which were plated on Luria–Bertani (LB) agar containing 100 μg ml^−1^ carbenicillin or kanamycin and grown at 37 °C overnight. *E. coli* transformants were grown in 5 ml LB medium containing 100 μg ml^−1^ carbenicillin or kanamycin at 37 °C overnight, followed by miniprep plasmid extraction (Qiagen), and were validated by Sanger sequencing. All biosynthetic genes^9,10^ with the exception of LovF-TE were codon-optimized for yeast expression and synthesized by Integrated DNA Technologies. The QS-21 biosynthetic pathway genes were directly inserted into the plasmid backbone for subcellular localization studies in *Nicotiana benthamiana*. All genes were assembled as expression cassettes in pESC plasmids or the plant binary expression vector pCaBGi for yeast and plant expression, respectively. All enzymes used in this study are listed in Supplementary Table 1.

### Strain construction

DNA integrating sequences were constructed using a previously described method^32^. Manufacturer protocols and standard recombinant DNA procedures were followed for DNA purification (Qiagen), DNA amplification (New England Biolabs, Q5 HighFidelity 2X Master Mix). All primers were designed using CASdesigner. In brief, DNA fragments to be integrated were PCR-amplified then co-transformed with a Cas9-based plasmid facilitating integration at the targeted locus. Alternatively, selection markers were integrated using homologous recombination. For transformations, a fresh overnight culture of parent yeast was inoculated into 25 ml 2×YPD in a 250-ml shake flask at an optical density at 600 nm (OD_600 nm_) of 0.2, and was incubated at 30 °C and 200 rpm until the OD_600 nm_ reached 1.0. Then, 5 OD of cells were collected by centrifuging for 2 min at 3,000*g*, and were washed with a half volume of H_2_O. The pellet was then resuspended with DNA fragments for integration (2 µg) and pCUT plasmid (0.25 µg), which was then mixed with transformation mix (260 µl of 50% PEG3350, 36 µl of 1 M LiOAc and 10 µl of ssDNA)^33^. The mixture was incubated at 42 °C for 30 min and the pellet was collected by centrifuging for 2 min at 3,000*g*. The pellets were then resuspended with 100 µl H_2_O and this was plated onto selective agar plates. The integration was validated by colony PCR and sequencing; the correct colonies were used for further engineering after pCUT plasmid curing. Oligonucleotides and codon-optimized gBlock gene fragments were obtained from Integrated DNA Technologies. Yeast culture media were purchased from BD, and all agar plates were obtained from Teknova. All strains constructed in this study are listed in Supplementary Table 2.

### In vivo production, extraction and analysis of QS-21 and its precursors

Strains were grown in 2 ml of yeast extract peptone dextrose (YPD, 4% D) medium for 48 h to reach OD_600 nm_ = 10–15, before being resuspended in 2 ml yeast extract peptone galactose (YPG, 4% G). All strains were incubated for 72 h in 24-deep-well plates at 30 °C and 200 rpm. YL-43 to YL-51 were supplemented with fresh YPG every 24 h. The medium was supplemented with 50–500 mg l^−1^ (*S*)-2-methylbutyric acid when culturing YL-42 to YL-47.

### β-amyrin production and GC–MS analysis

A single method was used to extract and quantify squalene and β-amyrin. Five hundred microlitres of culture medium in a microfuge tube was first treated with Zymolyase 100T (*Arthrobacter luteus*, AMSBIO) for 2 h at 37 °C before it was extracted with 500 µl ethyl acetate with bead-beating (3,800 rpm, 1 min × 2). Cholesterol was used as an internal standard. Organic and inorganic layers were separated by centrifugation at 12,000*g* for 1 min, and samples were extracted twice using cholesterol as an internal standard. Two hundred microlitres of the combined organic layer is derived by treatment with 200 µl of pyridine and 200 µl of BSTFA (Sigma-Aldrich) at 55 °C for 1 h. The derived sample was diluted in ethyl acetate before it was subjected to GC–MS (GC model 6890, MS model 5973 inert, Agilent). An aliquot of the sample (1 µl) was injected into a DB-WAX column (Agilent) operating at a helium flow rate of 1 ml min^−1^. The oven temperature was held at 80 °C for 4 min after injection and was then ramped to 280 °C at 20 °C min^−1^, held at 280 °C for 25 min, ramped to 300 °C at 20 °C min^−1^ and finally held at 300 °C for 5 min (total method of 45 min). The MS ion source was held at 300 °C throughout, with the quadrupole at 200 °C and the GC–MS transfer line at 280 °C. Full mass spectra were generated for metabolite identification by scanning within the *m*/*z* range of 40–440. Standard curves for target molecules were routinely run at the start and end of each batch of samples.

### Triterpenoid production and LC–MS analysis

The extraction and detection of erythrydiol (**2**) follow the procedure described for β-amyrin. For the rest of the triterpenoids, 200 µl of culture was collected in a microfuge tube before it was directly extracted with 800 µl methanol with bead-beating (3,800 rpm, 1 min × 2). The mixture was centrifuged at 12,000*g* for 1 min to separate the pellet. Two hundred microlitres of the supernatant was transferred into an Eppendorf tube, which was then evaporated in a vacuum concentrator at room temperature and the remainders were resuspended in 200 µl methanol. Finally, samples were filtered with Amicon Ultra 0.5-ml 3-kDa filter tubes or centrifuged at 15,000*g* for 5 min. Products were analysed using LC–MS (1260 Infinity II LC-MSD iQ, Agilent) equipped with a reverse phase C18 column (Kinetex 2.6 µm, 250 × 4.6 mm, XB-C18, Phenomenex). A 50-min isocratic method was performed with 10:90 of water (solvent A) and acetonitrile (solvent B) using a flow rate of 0.3 ml min^−1^. Full mass spectra were generated for metabolite identification by scanning within the *m*/*z* range of 300–600 in negative-ion mode. Data acquisition and analysis were performed using OpenLab CDS version 2.4 (Agilent).

### Production of glycosylated QA and LC–MS analysis

A similar extraction procedure was followed, by collecting 500 µl of culture and mixing with 500 µl methanol with bead-beating (3,800 rpm, 1 min × 2). Two hundred microlitres of the supernatant was evaporated and was resuspended in 200 µl of methanol before C28 glycosylation; otherwise, 800 µl of the supernatant was resuspended in 160 µl of methanol. Detection of glycosylated triterpenoids was performed using an LC-MSD iQ equipped with a Kinetex column 2.6 μm XB-C18 100 Å, 50 × 2.1 mm (Phenomenex) using the following parameters^9^: MS (ESI ionization, desolvation line temperature = 250 °C, nebulizing gas flow = 15 l min^−1^, heat block temperature = 400 °C, spray voltage positive 4.5 kV, negative −3.5 kV). Method: solvent A: (H_2_O + 0.1% formic acid); solvent B: (acetonitrile (CH_3_CN) + 0.1% formic acid). Injection volume: 10 µl. Gradient: 15% B from 0 to 1.5 min, 15% to 60% B from 1.5 to 26 min, 60% to 100% B from 26 to 26.5 min, 100% B from 26.5 to 28.5 min, 100% to 15% B from 28.5 to 29 min, 35% B from 29 to 30 min. The method was performed using a flow rate of 0.3 ml min^−1^. Full mass spectra were generated for metabolite identification by scanning within the *m*/*z* range of 400–1,350 in negative-ion mode. Data acquisition and analysis were performed using OpenLab CDS v.2.4 (Agilent). The production of target molecules was confirmed by co-elution with the purified standards previously reported^9^.

### Production of acylated molecules and QS-21, and LC–QTOF–MS analysis

A similar extraction procedure was followed, by collecting 500 µl of culture and mixing with 500 µl methanol with bead-beating (3,800 rpm, 1 min × 2). Eight hundred microlitres of the supernatant was evaporated and was resuspended in 40 µl methanol, which was then filtered with Amicon Ultra 0.5-ml 3-kDa filter tubes or centrifuged at 15,000*g* for 5 min. Detection of the acylated molecules and QS-21 was performed by LC–MS (Agilent 6545 for quadrupole time-of-flight (QTOF), Agilent) using the following parameters^10^: MS (ESI ionization, desolvation line temperature = 250 °C, nebulizing gas flow = 15 l min^−1^, heat block temperature = 400 °C, spray voltage positive 4.5 kV, negative −3.5 kV). Method: solvent A: (H_2_O + 0.1% formic acid); solvent B: (acetonitrile (CH_3_CN) + 0.1% formic acid). Injection volume: 10 µl. Gradient: 15% B from 0 to 0.75 min, 15% to 60% B from 0.75 to 13 min, 60% to 100% B from 13 to 13.25 min, 100% to 15% B from 13.25 to 14.5 min, 15% B from 14.5 to 16.5 min. The method was performed using a flow rate of 0.6 ml min^−1^ and a Kinetex column 2.6 μm XB-C18 100 Å, 50 × 2.1 mm (Phenomenex). Full mass spectra were generated for metabolite identification by scanning within the *m*/*z* range of 400–2,500 in negative-ion mode^19^. Analysis was performed using MassHunter Qualitative Analysis v.B.06.00 (Agilent). Note that the standard used to spike in the QS-21 sample was **18**-Xyl, which was generated in vitro using **18** with a terminal apiose on the C28 sugar chain. Because the molecules with a C28 terminal apiose or xylose co-elute, **18**-Xyl (C28 terminal apiose) was used to determine the elution time of **17**-Xyl (C28-terminal-xylose).

### Extraction of CoA from engineered yeast and LC–MS analysis

The extraction procedure was adapted from previous reports^28,34^. Specifically, 5 OD of cells were pelleted by centrifugation for 2 min at 4 °C at 3,000*g* and the supernatant was discarded. Cells were quenched and extracted by 100 μl of methanol: acetonitrile: 0.1% glacial acetic acid at a 45:45:10 ratio prechilled at −20 °C. The resuspended extracts were incubated on ice with intermittent vortexing for 15 min, followed by a 3-min centrifugation at 12,000*g* and 4 °C. The supernatant (10 µl) was injected for LC–MS analysis. Detection of CoA was performed using an LC-MSD iQ equipped with a Hypercarb column 5 μm, 250 Å, 150 × 1 mm (Thermo Fisher Scientific) using the following parameters: MS (ESI ionization, desolvation line temperature = 350 °C, nebulizing gas flow = 13 l min^−1^, spray voltage positive 4.5 kV, negative −6.0 kV). Method: solvent A: (H_2_O + 0.1% formic acid); solvent B: (acetonitrile (CH_3_CN) + 0.1% formic acid). Injection volume: 10 µl. Gradient: 2% B from 0 to 15 min, 2% to 90% B from 15 to 17 min, 90% to 20% B from 17 to 18 min, 2% B from 18 to 35 min. The method was performed using a flow rate of 0.1 ml min^−1^. Full mass spectra were generated for metabolite identification by scanning within the *m*/*z* range of 300–1,300 in negative-ion mode. Data acquisition and analysis were performed using OpenLab CDS v.2.4 (Agilent). The 2MB-CoA standard was synthesized according to a reported procedure^29^.

### Transient expression of fluorescent fusion proteins in tobacco plants

Leaves of four-week-old *N. benthamiana* plants were infiltrated following a procedure adapted from a previous study^35^. In brief, constructs assembled into binary vectors were transformed into the *Agrobacterium tumefaciens* strain GV3101. Transformed *Agrobacterium* strains were grown in LB with appropriate antibiotics at 30 °C, shaking at 200 rpm, to an OD_600 nm_ of 0.8–1.2. *Agrobacterium* cells were collected by centrifugation at 4,000*g* for 10 min at room temperature and resuspended in infiltration buffer (10 mM MES, 10 mM MgCl_2_ and 500 µM acetosyringone) to final OD_600 nm_ = 0.5. Cells were incubated in the infiltration buffer for 1 h with gentle shaking. *N. benthamiana* leaves were infiltrated with a 1-ml syringe with no needle attached by gently pressing the syringe to the abaxial side of the leaf while applying gentle pressure to the adaxial side. *N. benthamiana* plants were grown and maintained in a plant growth room at 25 °C in 16-h–8-h light–dark cycles with 50% humidity. Leaves were collected three days after infiltration.

### Reporting summary

Further information on research design is available in the Nature Portfolio Reporting Summary linked to this article.

## Online content

Any methods, additional references, Nature Portfolio reporting summaries, source data, extended data, supplementary information, acknowledgements, peer review information; details of author contributions and competing interests; and statements of data and code availability are available at 10.1038/s41586-024-07345-9.

### Supplementary information

Supplementary Information

Reporting Summary

### Source data

Source Data Figs. 2 and 3 and Source Data Extended Data Figs. 1–4, 6 and 10

## Data Availability

Strains and plasmids developed for this study (Supplementary Table 2), along with annotated sequences, have been deposited in the JBEI Registry (https://registry.jbei.org) and are physically available from the authors upon reasonable request. Contractual obligations from commercial partnerships prohibit us from distributing (by ourselves or through a third party) strains described in our manuscript to for-profit commercial entities. However, we provide extensive genotypic descriptions of our strains, fully annotated DNA sequences and detailed methods that will enable others to build on our work. Strains will be provided to nonprofit, government or academic laboratories and institutions. Source data are provided with this paper.
