# Supplementary material for: Complete biosynthesis of QS-21 in engineered yeast
Source: Nature. 2024 May 8;629(8013):937–44. doi: 10.1038/s41586-024-07345-9 (PMC11111400; doi:10.1038/s41586-024-07345-9)
Supplement: Supplementary file 1 — Supplementary Information [file 41586_2024_7345_MOESM1_ESM.pdf]

nature portfolio

https://doi.org/10.1038/s41586-024-07345-9

Supplementary information

Complete biosynthesis of QS-21 in engineered yeast

In the format provided by the authors and unedited

Supplementary Information

Complete biosynthesis of QS-21 in engineered yeast

Yuzhong Liu,[1,2] Xixi Zhao,[1,2] Fei Gan,[1,2] Xiaoyue Chen,[2,3,4] Kai Deng,[2,12] Samantha A. Crowe,[1,2,5] Graham A. Hudson,[1,2] Michael S. Belcher,[2,3,4] Matthias Schmidt,[2,6,7] Maria C. T. Astolfi,[2,8] Suzanne Kosina,[4] Bo Pang,[1,2] Minglong Shao,[2,6] Jing Yin,[2,6] Sasilada Sirirungruang,[2,3,4,9] Anthony T. Iavarone,[1] James Reed,[10] Laetitia B. B. Martin,[10] Amr El-Demerdash,[10,11] Shingo Kikuchi,[10] Rajesh Chandra Misra,[10] Xiaomeng Liang,[2,6] Michael J. Cronce,[2,8] Xiulai Chen,[2,6] Chunjun Zhan,[2,6] Ramu Kakumanu,[2,6] Edward E. K. Baidoo,[2,6] Yan Chen,[2,6] Christopher J. Petzold,[2,6] Trent Northen,[2,4] Anne Osbourn,[10] Henrik Scheller,[2,3,4] Jay D. Keasling[1,2,5,6,8,13,14*]

[1] California Institute of Quantitative Biosciences (QB3), University of California, Berkeley, CA, USA
[2] Joint BioEnergy Institute, Emeryville, CA, USA
[3] Department of Plant and Microbial Biology, University of California, Berkeley, CA 94720, USA.
[4] Environmental Genomics and Systems Biology Division, Lawrence Berkeley National Laboratory, Berkeley, CA, USA
[5] Department of Chemical & Biomolecular Engineering, University of California, Berkeley, CA, USA
[6] Division of Biological Systems and Engineering, Lawrence Berkeley National Laboratory, Berkeley, CA, USA
[7] Institute of Applied Microbiology (iAMB), Aachen Biology and Biotechnology (ABBt), RWTH Aachen University, Aachen, Germany
[8] Departments of Bioengineering, University of California, Berkeley, CA, USA
[9] Center for Biomolecular Structure, Function and Application, Suranaree University of Technology, Nakhon Ratchasima, Thailand.
[10] John Innes Centre, Norwich Research Park, Norwich, NR4 7UH, UK.
[11] Department of Chemistry, Faculty of Sciences, Mansoura University, Mansoura 35516, Egypt.
[12] Department of Biomaterials and Biomanufacturing, Sandia National Laboratories, Livermore, CA, USA.
[13] Center for Biosustainability, Danish Technical University, Lyngby, DK
[14] Center for Synthetic Biochemistry, Shenzhen Institutes for Advanced Technologies, Shenzhen, China

*Correspondence: keasling@berkeley.edu (J.D.K)

Table of Content

Supplementary Table 1. List of heterologous genes for QS-21 biosynthesis S3

Supplementary Table 2. List of yeast strains used in this work S6

Supplementary Table 3. List of structures and IUPAC nomenclature of intermediates......... S10

Supplementary Table 4. List of chemicals used in this work S14

Supplementary Figure 1. LC-MS traces of (5) produced in YL-3 .... S15

Supplementary Section 1. Gene discovery of MSBP in *Saponaria vaccaria* S16

Supplementary Table 5. *Arabidopsis thaliana* MSBP homologs in *Saponaria vaccaria*......... S16

Supplementary Figure 2. Transcript expression profiles in *Saponaria vaccaria* S17

Supplementary Figure 3. LC-MS traces of (7) produced in YL-17 .... S19

Supplementary Figure 4. LC-MS traces of (8) produced in YL-18 .... S20

Supplementary Figure 5. LC-MS traces of (9) produced in YL-20 .... S21

Supplementary Figure 6. LC-MS traces of (11) and (12) produced in YL-27 and YL-29.......... S23

Supplementary Figure 7. LC-MS traces of (13) and (14) produced in YL-33 and YL-34.......... S24

Supplementary Figure 8. Subcellular localization studies of XylT4 .... S26

Supplementary Figure 9. LC-MS traces of QS-21-Api produced in YL-47 .... S28

Supplementary Section 2. QS-21 production and purification S29

Supplementary Section 3. Tandem mass spectrometry S30

Supplementary Table 6. LC-MS parameters for QS-21 MS1 and MS2 measurement S31

Supplementary Table 7. MS2 fragment ion masses and their corresponding intensities S33

Supplementary Figure 10. LC-MS traces of QS-21 produced in yeast S34

Supplementary Section 4. Calculation of QS-21 content in yeast and in plant..................... S35

Supplementary Tables

Supplementary Table 1. List of heterologous genes for QS-21 biosynthesis

Enzyme	Origin	Short Name	GenBank or reference
β-amyrin synthase	*Artemisia annua* L.	AaBAS	ACA13386.1
	Arabidopsis thaliana (L.) Heynh.	AtBAS	BAG82628.1
	Glycyrrhiza glabra L.	GgBAS	QBC36434.1
	Saponaria vaccaria L.	SvBAS	ABK76265.1
ERG20	*Saccharomyces cerevisiae*	ERG20	CAA89462.1
ERG1	*Saccharomyces cerevisiae*	ERG1	CAA97201.1
NADPH-cytochrome P450 reductase	*Arabidopsis thaliana*	ATR1	CAA46814.1
C28 oxidase (CYP716A224)	*Quillaja saponaria*	QsC28 oxidase	Reed et al.[1]
C23 oxidase (CYP714E52)	*Quillaja saponaria*	QsC23 oxidase	Reed et al.[1]
cytochrome b5 reductase	*Quillaja saponaria*	Qsb_5	Reed et al.[1]
C16 oxidase (CYP716A297)	*Quillaja saponaria*	QsC16 oxidase	Reed et al.[1]
		TMD_{C28}-C16 or QsC28C16	This work
Membrane steroid binding protein	*Arabidopsis thaliana*	AtMSBP1	OAO91144.1
	Saponaria vaccaria	SvMSBP1	This study
	Saponaria vaccaria	SvMSBP2	This study
UDP-glucose dehydrogenase	*Arabidopsis thaliana*	AtUGD1	AEE30705.1
	Arabidopsis thaliana	$AtUGD1_{A101L}$	This study
C3-UDP-glucuronic acid transferase	*Quillaja saponaria*	CSLM1 or CSLG1	Reed et al.[1]
	Quillaja saponaria	CSLM2 or CSLG2	Reed et al.[1]
C3-UDP-galactose transferase (UGT73CU3)	*Quillaja saponaria*	C3GalT	Reed et al.[1]
UDP-xylose synthase 3	*Arabidopsis thaliana*	AtUXS3 or ATUXS	AED97167.1
C3-UDP-xylose transferase (UGT73CX1)	*Quillaja saponaria*	C3XylT	Reed et al.[1]

C3-UDP-rhamnose transferase (UGT73CX2)	*Quillaja saponaria*	C3RhaT	Reed et al.[1]
UDP-glucose 4,6-dehydratase	*Saponaria vaccaria*	SvUG46DH	Chen et al.[2]
Short chain dehydrogenase/reductase	*Quillaja saponaria*	QsFucSyn	Reed et al.[1]
C28-UDP-fucose transferase (UGT74BX1)	*Quillaja saponaria*	C28FucT	Reed et al.[1]
Trifunctional UDP-glucose 4,6-dehydratase/UDP-4-keto-6-deoxy-D-glucose 3,5-epimerase/UDP-4-keto-L-rhamnose-reductase 2	*Arabidopsis thaliana*	AtRHM2	CAD92667.1
C28-UDP-rhamnose transferase (UGT91AR1)	*Quillaja saponaria*	C28RhaT	Reed et al.[1]
C28-UDP-xylose transferase-3 (UGT91AQ1)	*Quillaja saponaria*	C28XylT3	Reed et al.[1]
C28-UDP-xylose transferase-4 (UGT73CY3)	*Quillaja saponaria*	C28XylT4	Reed et al.[1]
UDP-D-apiose/UDP-D-xylose synthase 1	*Arabidopsis thaliana*	AtAXS1	AAR14687.1
C28-UDP-xylose transferase-4 (UGT73CY2)	*Quillaja saponaria*	C28ApiT4	Reed et al.[1]
Short-chain fatty acid CoA ligase	*Quillaja saponaria*	QsCCL	Martin et al.[3]
Chalcone-synthase like enzymes	*Quillaja saponaria*	PKS4 or ChSD	Martin et al.[3]
	Quillaja saponaria	PKS5 or ChSE	Martin et al.[3]
Keto reductases	*Quillaja saponaria*	KR1 or KR11	Martin et al.[3]
	Quillaja saponaria	KR2 or KR23	Martin et al.[3]
Acyl transferase 1	*Quillaja saponaria*	ACT2 or DMOT9	Martin et al.[3]
Acyl transferase 2	*Quillaja saponaria*	ACT3 or DMOT4	Martin et al.[3]
Bifunctional UDP-D-glucose/UDP-D-galactose 4-epimerase 1	*Arabidopsis thaliana*	AtUGE1 or AtUXE	CAA90941.1

Reversibly glycosylated polypeptide 1	*Arabidopsis thaliana*	AtRGP1	AAC50000.1
UDP-arabinofuranose transferase (UGT73CZ2)	*Quillaja saponaria*	ArafT	Martin et al.[3]
4'-Phospho-pantetheinyl transferase	*Aspergillus nidulans FGSC A4*	npgA	AAF12814.1
Lovastatin biosynthesis cluster protein F	*Aspergillus terreus*	LovF	AAD34559.1
Thioesterase domain from 6-Deoxy-erythronolide Synthase	*Saccharopolyspora erythraea*	DEBS-TE	CAA39583.1

Supplementary Table 2. List of yeast strains in this study

Strain	Parent strain	Genotype	Note	Reference
Cen.pk2-1c		MATa; his3-1; leu2-3_112; ura:3-52; trp1-2; MAL2-8c; SUC2	Wild-type strain	Euroscarf
GTy23	Cen.pk2-1c	erg9::KanMX_pCTR3-ERG9, leu2-3, 112::His3MX6_pGal1-ERG19, pGal10-ERG8, ura3-52::URA3_pGal1-mvaS, pGal10- mvaE, his3Δ1::hphMX4_pGal1-ERG12, pGal10-IDI1	Yeast strain with an upregulated mevalonate pathway	Wong et al.[4]
JWy601	GTy23	ura3-52 prototrophy removed for use of Cas9 system	Yeast strain with an upregulated mevalonate pathway	Wong et al.[4]
MLY-AabAS	JWy601	308a::pGal1-AabAS	β-amyrin (**1**) producing strain	This study
MLY-AtbAS	JWy601	308a::pGal1- AtbAS	β-amyrin (**1**) producing strain	This study
MLY-GgbAS	JWy601	308a::pGal1-GgbAS	β-amyrin (**1**) producing strain	This study
MLY-SvbAS	JWy601	308a::pGal1-SvbAS	β-amyrin (**1**) producing strain	This study
MLY-01	JWy601	1114a::pGal1-ERG20, 1622b:: pGal1-ERG1, 308a::pGal1-SvBAS	optomized β-amyrin (**1**) producing strain	This study
YL-1	MLY-01	607c::pGal1-QsC28, pGal10- ATR1	Oleanolic acid (**3**)	This study
YL-2	YL-1	416d::pGal1-QsC23	Hederagenin (**4**)	This study
YL-3	YL-1	416d::pGal1-QsC23, pGal10-Qsb5	Gypsogenin (**5**)	This study
YL-4	YL-3	911b::pGal1-QsTMD$_{C28}$-C16	Quillaic acid (QA, **6**)	This study
YL-5	YL-4	805a::pGal1-SvMSBP1	Quillaic acid (QA, **6**)	This study
YL-6	YL-4	805a::pGal1-SvMSBP2	Quillaic acid (QA, **6**)	This study
YL-7	YL-4	805a::pGal1-AtMSBP1	Quillaic acid (QA, **6**)	This study
YL-8	YL-4	805a::pGal1-QsC28	Quillaic acid (QA, **6**)	This study
YL-9	YL-4	805a::pGal1-QsC23	Quillaic acid (QA, **6**)	This study
YL-10	YL-4	805a::pGal1-QsTMD$_{C28}$-C16	Quillaic acid (QA, **6**)	This study

YL-11	YL-4	805a::pGal1-QsC28, pGal10- ATR1	Quillaic acid (QA, **6**)	This study
YL-12	YL-4	805a::pGal1-QsTMD$_{C28}$-C16, pGal10-SvMSBP1	Quillaic acid (QA, **6**)	This study
YL-13	YL-5	1414a::pGal1-QsC28, pGal10-ATR1	Quillaic acid (QA, **6**)	This study
YL-14	YL-5	δ::pGal1-QsC28, pGal10-ATR1	Quillaic acid (QA, **6**)	This study
YL-15	YL-5	1414a::pGal1-QsC28, pGal10-ATR1, pGal1-QsC23, pGal10-Qsb5, pGal1-QsTMD$_{C28}$-C16, pGal10-SvMSBP1	Quillaic acid optimization (QA, **6**)	This study
YL-16	YL-15	106a::LEU2-3_pGal1-QsCSLM1, pGal10-AtUGD	QA-C3-GlcA (**7**)	This study
YL-17	YL-15	106a::LEU2-3_pGal1-QsCSLM2, pGal10-AtUGD	QA-C3-GlcA (**7**)	This study
YL-18	YL-15	106a::LEU2-3_pGal1-QsCSLM2, pGal10-AtUGD, pGal1-C3GalT	QA-C3-GlcA-Gal (**8**)	This study
YL-19	YL-15	106a::LEU2-3_pGal1-QsCSLM2, pGal10-AtUGD$_{A101L}$, pGal1-QsGalT, pGal1-C3RhaT, pGal10-AtRHM2	QA-C3-GlcA-Gal-Rha (**22**)	This study
YL-20	YL-15	106a::LEU2-3_pGal1-QsCSLM2, pGal10-AtUGD$_{A101L}$, pGal1-QsGalT, pGal1-C3XylT, pGal10-AtUXS	QA-C3-GlcA-Gal-Xyl (**9**)	This study
YL-24	YL-19	1021b::URA_pGal1-QsFucSyn, pGal10-SvUG46DH, pGal1-C28FucT	QA-C3-GlcA-Gal-Rha-C28-Fuc (**23**)	This study
YL-25	YL-20	1021b::URA_pGal1-QsFucSyn, pGal10-SvUG46DH, pGal1-C28FucT	QA-C3-GlcA-Gal-Xyl-C28-Fuc (**10**)	This study
YL-26	YL-19	1021b::URA_pGal1-QsFucSyn, pGal10-SvUG46DH, pGal1-C28FucT, pGal1-C28RhaT, pGal10-AtRHM2	QA-C3-GlcA-Gal-Rha-C28-Fuc-Rha (**24**)	This study
YL-27	YL-20	1021b::URA_pGal1- QsFucSyn, pGal10-SvUG46DH, pGal1-C28FucT, pGal1-C28RhaT, pGal10-AtRHM2	QA-C3-GlcA-Gal-Xyl-C28-Fuc-Rha (**11**)	This study
YL-28	YL-26	208a::NAT_pGal1-C28XylT3, pGal10-AtUXS	QA-C3-GlcA-Gal-Rha-C28-Fuc-Rha-Xyl (**25**)	This study
YL-29	YL-27	208a::NAT_pGal1-C28XylT3	QA-C3-GlcA-Gal-Xyl-C28-Fuc-Rha-Xyl (**12**)	This study

YL-30	YL-27	208a::NAT_pGal1-C28XylT3, pGal10-AtUXS	QA-C3-GlcA-Gal-Xyl-C28-Fuc-Rha-Xyl (**12**)	This study
YL-31	YL-28	1206a::TRP_pGal1-C28XylT4, pGal10-AtUXS	QA-C3-GlcA-Gal-Rha-C28-Fuc-Rha-Xyl-Xyl (**26**)	This study
YL-32	YL-28	1206a::TRP_pGal1-C28ApiT4, pGal10-AtAXS	QA-C3-GlcA-Gal-Rha-C28-Fuc-Rha-Xyl-Api (**27**)	This study
YL-33	YL-30	1206a::TRP_pGal1-C28XylT4, pGal10-AtUXS	QA-C3-GlcA-Gal-Xyl-C28-Fuc-Rha-Xyl-Xyl (**13**)	This study
YL-34	YL-30	1206a::TRP_pGal1-C28ApiT4, pGal10-AtAXS	QA-C3-GlcA-Gal-Xyl-C28-Fuc-Rha-Xyl-Api (**14**)	This study
YL-38	YL-30	1206a::TRP_pGal1-SUMOC28XylT4, pGal10-AtUXS	QA-C3-GlcA-Gal-Xyl-C28-Fuc-Rha-Xyl-Xyl (**13**)	This study
YL-39	YL-30	1206a::TRP_pGal1-MBPC28XylT4, pGal10-AtUXS	QA-C3-GlcA-Gal-Xyl-C28-Fuc-Rha-Xyl-Xyl (**13**)	This study
YL-40	YL-30	1206a::TRP_pGal1-DsREDC28XylT4, pGal10-AtUXS	QA-C3-GlcA-Gal-Xyl-C28-Fuc-Rha-Xyl-Xyl (**13**)	This study
YL-42	YL-30	YPRdelta::pGal1-QsPKS4, pGal10-PKS5, pGal1-KR1, pGal10-KR2, pGal1-QsCCL, pGal10-ACT2	QA-C3-GlcA-Gal-Xyl-C28-Fuc-Rha-Xyl-C9 (**19**)	This study
YL-43	YL-42	1206a::TRP_pGal1-C28XylT4	QA-C3-GlcA-Gal-Xyl-C28-Fuc-Rha-Xyl-Xyl-C9 (**15**)	This study
YL-44	YL-42	1206a::TRP_pGal1-ACT3	QA-C3-GlcA-Gal-Xyl-C28-Fuc-Rha-Xyl-C18 (**20**)	This study
YL-45	YL-42	1206a::TRP_pGal10- ACT3, pGal1-C28XylT4	QA-C3-GlcA-Gal-Xyl-C28-Fuc-Rha-Xyl-Xyl-C18 (**17**)	This study
YL-46	YL-42	1206a::TRP_pGal10-AtUXS, pGal1-C28XylT4, pGal10-AtUGE1, pGal1-AtRGP1, pGal10-ACT3, pGal1-ArafT	QS-21-Xyl	This study

YL-47	YL-42	1206a::TRP_pGal10-AtAXS, pGal1-C28ApiT4, pGal10-AtUGE1, pGal1-AtRGP1, pGal10-ACT3, pGal1-ArafT	QS-21-Api	This study
YL-50	YL-46	720a::ZeoR_pADH2-ngpA, pGal1-LovF-TE	QS-21-Xyl	This study
YL-51	YL-47	720a::ZeoR_pADH2-ngpA, pGal1-LovF-TE	QS-21-Api	This study
YL-QsCCL	Cen.pk2-1c	pESC-LEU::pGal10-QsCCL	Wild-type strain overexpressing QsCCL from plasmid	This study
YL-npgA	Cen.pk2-1c	δ::pADH-npgA	Wild-type strain overexpressing npgA	This study
YL-PKS	YL-npgA	pESC-LEU::pGal10-QsCCL, pGal1-LovF-TE	2-MB production from LovF-TE	This study

Supplementary Table 3. Structures and IUPAC nomenclature of glycosylated intermediates

Number	Trivial name	IUPAC nomenclature	Structure
**7**	QA-C3-GlcA	3-*O*-{β-D-glucopyranosiduronic acid}-quillaic acid	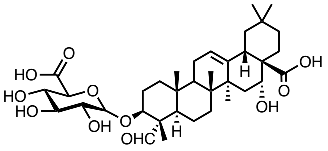
**8**	QA-C3-GlcA-Gal	3-*O*-{β-D-galacto-pyranosyl-(1→2)-b-D-glucopyranosiduronic acid}-quillaic acid	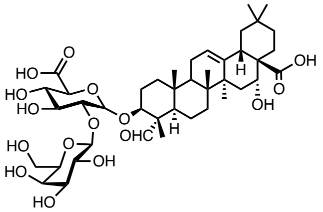
**9**	QA-C3-GlcA-Gal-Xyl	3-*O*-{β-D-xylopyranosyl-(1→3)-[β-D-galactopyranosyl-(1→2)]-β-D-glucopyranosiduronic acid}-quillaic acid	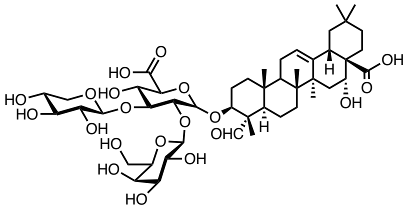
**10**	QA-C3-GlcA-Gal-Xyl-C28-Fuc	3-*O*-{β-D-xylopyranosyl-(1→3)-[β-D-galactopyranosyl-(1→2)]-β-D-glucopyranosiduronic acid}-28-*O*-{β-D-fucopyranosiduronic ester}-quillaic acid	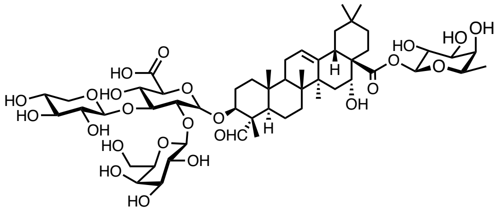

**11**	QA-C3-GlcA-Gal-Xyl-C28-Fuc-Rha	3-*O*-{β-D-xylopyranosyl-(1→3)-[β-D-galactopyranosyl-(1→2)]- β-D-glucopyranosiduronic acid}-28-*O*-{α-L-rha-nopyranosyl-(1→2)- β-D-fucopyranosiduronic ester}-quillaic acid	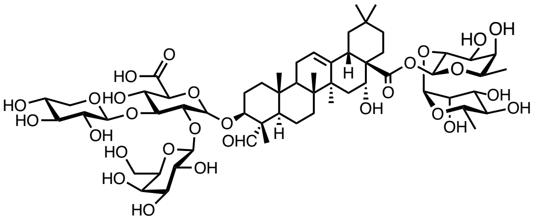
**12**	QA-C3-GlcA-Gal-Xyl-C28-Fuc-Rha-Xyl	3-*O*-{β-D-xylopyranosyl-(1→3)-[β-D-galactopyranosyl-(1→2)]-β-D-glucopyranosiduronic acid}-28-*O*-{β-D-xylopyranosyl-(1→4)-α-L-rhamnopyranosyl-(1→2)- β-D-fucopyrano-siduronic ester}-quillaic acid	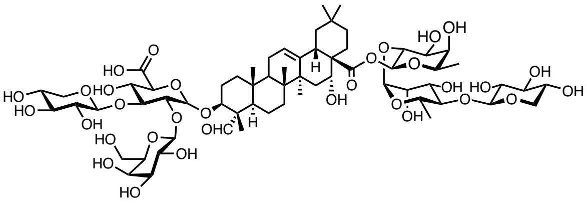
**13**	QA-C3-GlcA-Gal-Xyl-C28-Fuc-Rha-Xyl-Xyl	3-*O*-{β-D-xylopyranosyl-(1→3)-[β-D-galactopyranosyl-(1→2)]-β-D-glucopyranosiduronic acid}-28-*O*-{β-D-xylopyranosyl-(1→4)-β-D-xylopyranosyl-(1→4)-α-L-rhamnopyranosyl-(1→2)-β-D-fucopyrano-siduronic ester}-quillaic acid	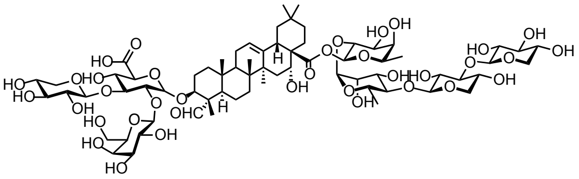

**14**	QA-C3-GlcA-Gal-Xyl-C28-Fuc-Rha-Xyl-Api	3-*O*-{β-D-xylopyranosyl-(1→3)-[β-D-galactopyranosyl-(1→2)]-β-D-glucopyranosiduronic acid}-28-*O*-{β-D-apiofuranosyl-(1→3)-β-D-xylopyranosyl-(1→4)-α-L-rhamnopyranosyl-(1→2)-β-D-fucopyrano-siduronic ester}-quillaic acid	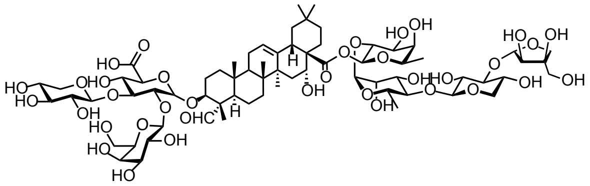
**22**	QA-C3-GlcA-Gal-Rha	3-*O*-{α-L-rhamnopyranosyl-(1→3)-[β-D-galactopyranosyl-(1→2)]-β-D-glucopyranosiduronic acid}-quillaic acid	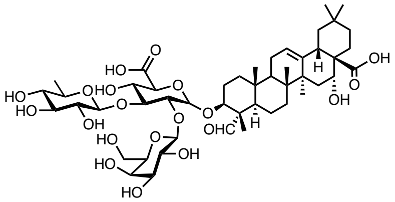
**23**	QA-C3-GlcA-Gal-Rha-C28-Fuc	3-*O*-{α-L-rhamnopyranosyl-(1→3)-[β-D-galactopyranosyl-(1→2)]-β-D-glucopyranosiduronic acid}-28-*O*-{β-D-fucopyranosiduronic ester}-quillaic acid	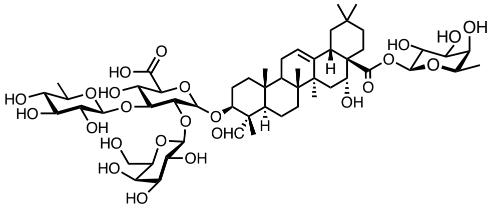
**24**	QA-C3-GlcA-Gal-Rha-C28-Fuc-Rha	3-*O*-{a-L-rhamnopyranosyl-(1→3)-[β-D-galactopyranosyl-(1→2)]-β-D-glucopyranosiduronic acid}-28-*O*-{α-L-rha-nopyranosyl-(1→2)-β-D-fucopyranosiduronic ester}-quillaic acid	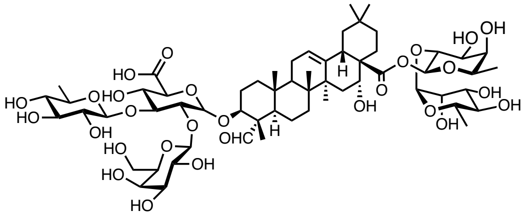

**25**	QA-C3-GlcA-Gal-Rha-C28-Fuc-Rha-Xyl	3-*O*-{α-L-rhamnopyranosyl-(1→3)-[β-D-galactopyranosyl-(1→2)]-β-D-glucopyranosiduronic acid}-28-*O*-{β-D-xylopyranosyl-(1→4)-a-L-rhamnopyranosyl-(1→2)-β-D-fucopyrano-siduronic ester}-quillaic acid	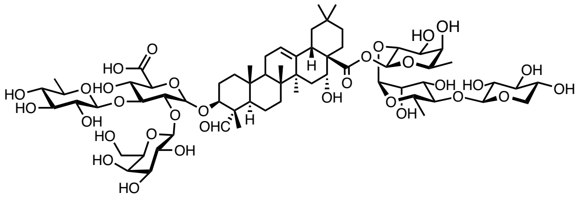
**26**	QA-C3-GlcA-Gal-Rha-C28-Fuc-Rha-Xyl-Xyl	3-*O*-{a-L-rhamnopyranosyl-(1→3)-[β-D-galactopyranosyl-(1→2)]-β-D-glucopyranosiduronic acid}-28-*O*-{β-D-xylopyranosyl-(1→4)-β-D-xylopyranosyl-(1→4)-α-L-rha-nopyranosyl-(1→2)-β-D-fucopyranosiduronic ester}-quillaic acid	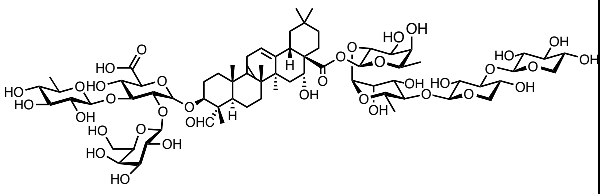
**27**	QA-C3-GlcA-Gal-Rha-C28-Fuc-Rha-Xyl-Api	3-*O*-{α-L-rhamnopyranosyl-(1→3)-[β-D-galactopyranosyl-(1→2)]-β-D-glucopyranosiduronic acid}-28-*O*-{β-D-apiofuranosyl-(1→3)-β-D-xylopyranosyl-(1→4)-α-a-L-rhamnopyranosyl-(1→2)-β-D-fucopyrano-siduronic ester}-quillaic acid	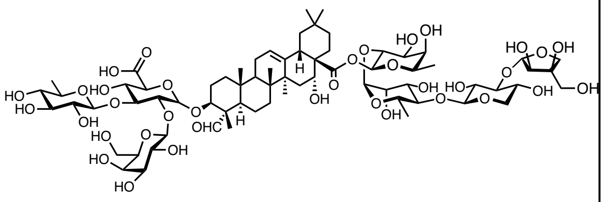

Supplementary Table 4. List of chemicals for feeding or analytical standards

Chemical	CAS #	Source supplier	Catalog #
Squalene	111-02-4	Sigma Aldrich	442785
β-amyrin (**1**)	559-70-6	BIOSYNTH	FA16227
Erythrodiol (**2**)	545-48-2	Sigma Aldrich	09258
Oleanolic acid (**3**)	508-02-1	AK Scientific	C507
Hederagenin (**4**)	465-99-6	MedChemExpress	HY-N0256/CS-5454
Gypsogenin (**5**)	639-14-5	BIOSYNTH	FG74392
Gypsogenic acid	5143-05-5	Santa Cruz Biotechnology	Sc-490170
Echinocystic acid	510-30-5	BIOSYNTH	FE65561
Caulophyllogenin	52936-64-8	BIOSYNTH	FC65490
Quillaic acid (**6**)	631-01-6	BIOSYNTH	FQ74391
QA-C3-GlcA (**7**)	NA	John Innes Centre	QA-mono[1]
QA-C3-GlcA-Gal (**8**)	NA	John Innes Centre	QA-di[1]
QA-C3-GlcA-Gal-Xyl (**9**)	NA	John Innes Centre	TriX[1]
QA-C3-GlcA-Gal-Rha (**22**)	NA	John Innes Centre	TriR[1]
QA-C3-GlcA-Gal-Rha-C28-Fuc (**23**)	NA	John Innes Centre	TriR-F[1]
QA-C3-GlcA-Gal-Rha-C28-Fuc-Rha (**24**)	NA	John Innes Centre	TriR-FR[1]
QA-C3-GlcA-Gal-Rha-C28-Fuc-Rha-Xyl (**25**)	NA	John Innes Centre	TriR-FRX[1]
QA-C3-GlcA-Gal-Rha-C28-Fuc-Rha-Xyl-Xyl (**26**)	NA	John Innes Centre	TriR-FRXX[1]
QA-C3-GlcA-Gal-Rha-C28-Fuc-Rha-Xyl-Api (**27**)	NA	John Innes Centre	TriR-FRXA[1]
QA-C3-GlcA-Gal-Xyl-C28-Fuc-Rha-Xyl-C9 (**19**)	NA	John Innes Centre	Tobacco extract[3]
QA-C3-GlcA-Gal-Rha-C28-Fuc-Rha-Xyl-Api-C18-Xyl (**18+Xyl**)	NA	John Innes Centre	Generated *in vitro*[3]
QS-21	141256-04-4	GlaxoSmithKline	
(*S*)-(+)-2-Methylbutyric acid	1730-91-2	Sigma Aldrich	245526

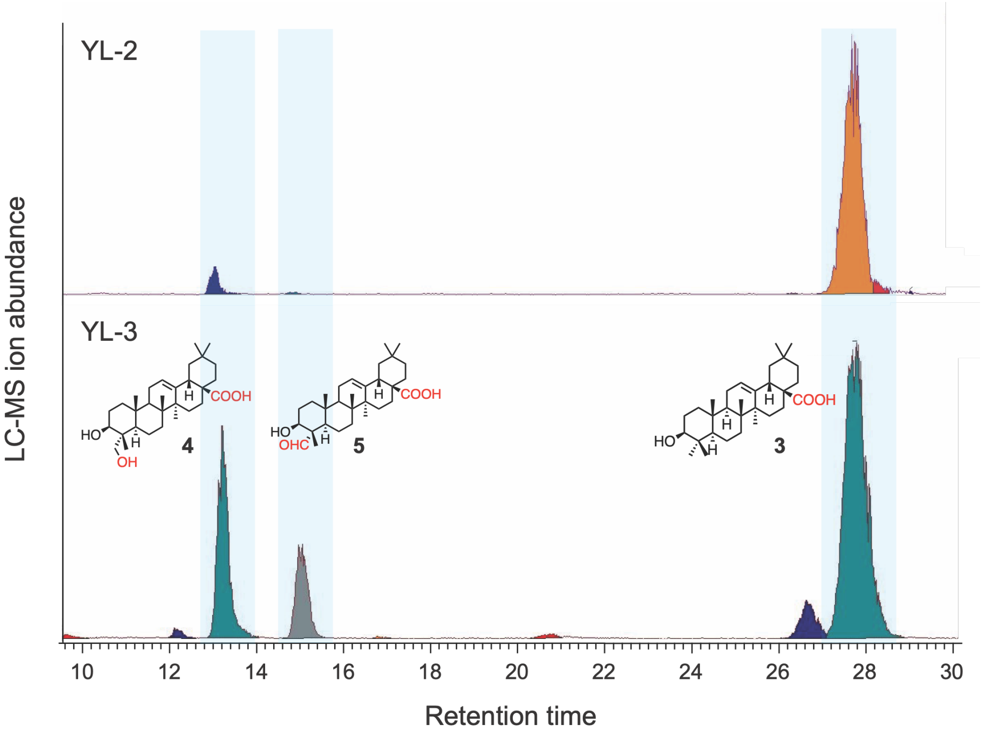

Supplementary Fig. 1 LC-MS traces of the extract from engineered yeasts. In the absence of the *Quillaja* native cytochrome b5 (Qsb$_5$), C23 position can only be oxidized to a hydroxyl group to form **4** in YL-2 at approximately 13.2 min retention time. When Qsb$_5$ was co-expressed in YL-3, the C23 hydroxyl group can be further oxidized to the desired aldehyde in **5**.

Gene discovery of MSBP proteins

Identification of the target mass of vaccaroside E, segetoside I, and segetoside I Ac by LC-MS in different organs of *S. vaccaria* suggested that saponins were present in roots, stems, leaves, and flowers.[2] Since cytochrome P450s (CYPs) are important enzymes involved in the saponin biosynthetic pathway and MSBPs are known to co-express with P450s, leaves and flowers were both collected and analyzed to serve the purpose of cross validation. Genes encoding MSBP homologs to *Arabidopsis thaliana* (At) have been identified in *Saponaria vaccaria* (Sv) transcriptome by sequence similarity search using algorithm tBLASTn. Amino acid sequences of MSBPs from At were submitted in a database of Sv transcriptome (prepared in-house) for comparison with translated DNA sequences of all genes in the transcriptome. Similar sequences were selected based on sequence identity (last column of **Supplementary Table 5**) and the significance of sequence match (third column of **Supplementary Table 5**). The results are summarized in Supplementary Table 5 below. The average expression levels of the different homologs identified in Supplementary Table 5 were also analyzed in leaves and flowers of *Saponaria vaccaria* (see **Supplementary Fig. 2**).

Supplementary Table 5. ***Arabidopsis thaliana*** **(At) MSBP homologs in** ***Saponaria vaccaria*** **(Sv)**

Query	Sv subject*	Sv subject length (bp)	E-value	% Identity
AtMSBP1	PB.394.2	695	6.58E-70	61.86
	PB.392.2	930	6.12E-69	61.86
	PB.394.1	1000	1.57E-68	61.86
	PB.393.1**	1203	8.72E-67	58.636
	PB.16084.1	960	3.93E-46	52.381
	PB.16084.2**	1429	9.84E-45	52.381
	PB.38275.1	1014	1.04E-13	40.244
AtMSBP2	PB.394.2	695	1E-80	72.105
	PB.393.1**	1203	7.7E-80	71.429
	PB.392.2	930	1.08E-79	72.105
	PB.394.1	1000	1.74E-79	72.105
	PB.16084.1	960	1.31E-53	53.846
	PB.16084.2**	1429	4.76E-52	53.846
	PB.38275.1	1014	1.6E-18	38.614

*Transcript names

**The longest 2 sequences (also showing the highest expression level in leaves and flowers in Supplementary Fig. 1) were selected for functional test in yeast (as described in the below Section 1.2.5).

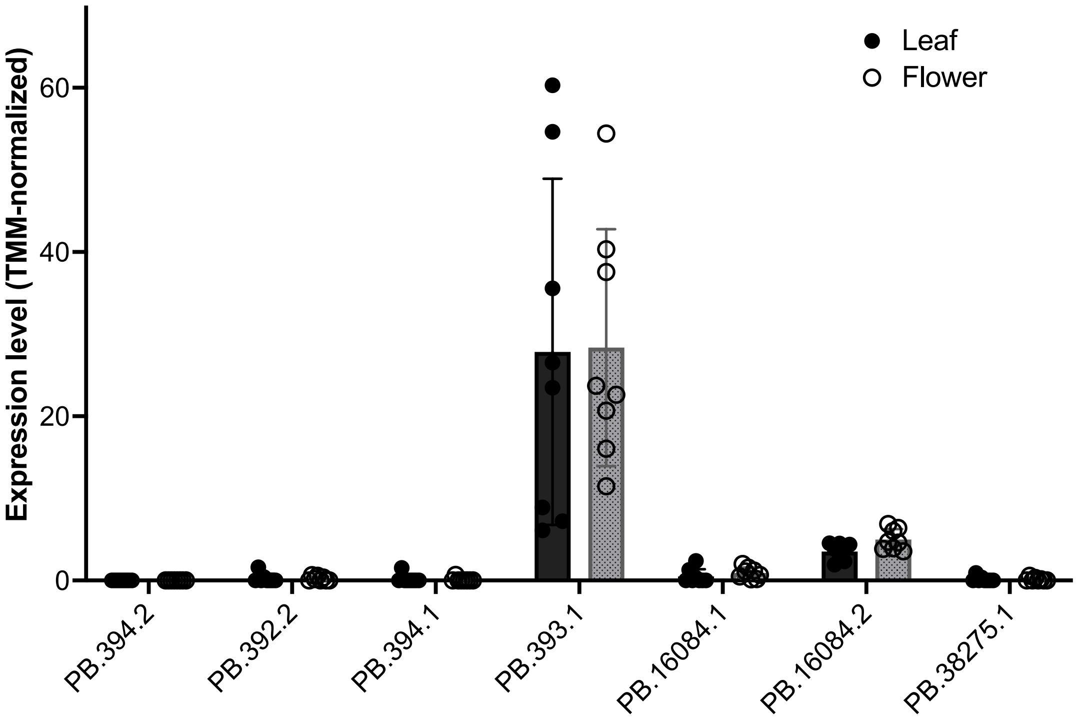

Supplementary Fig. 2 Transcript expression profile of AtMSBP homologs in leaves and flowers of *S. vaccaria*. Data are mean ± s.d.; n = 3 biologically independent samples.

***Arabidopsis thaliana* (At) MSBP homologs in *Saponaria vaccaria* (Sv)**

>PB.393.1 (CDS)

ATGTCAATAGAAATATGGGAAACACTGAAAGAAGCAATAACAACATACACAGGACTATCACCAACACTA
TTCTTCACAATAGTTGCACTTAGTCTTGCATTCTACCATGCTGTTTTTGGGTTATTTGGTTCATCATCATCA
TCATCATCATCATCATCATCAAATAGCCACCAAAATCCAAGGAATTTTGTTGAGGAAAGTGAGCCTTTAC
CACCACCTGTGCAACTTGGTGAGATTACTGAGGATGACTTGAAAAACTATGATGGCTCTGATTCTAAGA
AGCCTTTGCTTATGGCCATTAAAGGCCAGATCTATGATGTTTCCCAAAGCAGGATGTTTTATGGTCCAGG
TGGGCCATATGCATTGTTTGCAGGAAAAGATGCAAGCAGAGCTCTGGCGAAGATGTCGTTTGAGGATA
AAGATCTGACAGGCGATATCTCTGGTCTCGGTCCATTCGAACTCGAGGCCTTACAGGACTGGGAGTACA
AGTTCATGAGTAAATACGTCAAGGTCGGAACTATCAAAAAGGATGCTCCTCCCAGTGACGCACCGTCTC
CTAGTGAACCCTCTGAAGCTGCTGATGTTACAGACAGGGAAGCTCCCAAACACGCAGAAGATGGCCCAG
CCGAGACAGTAGAACCATCCTCTGTTGGTGATGCCGAGAAAAAAGAAGAGTAA

>PB.393.1 (aa)

MSIEIWETLKEAITTYTGLSPTLFFTIVALSLAFYHAVFGLFGSSSSSSSSSSSNSHQNPRNFVEESEPLPPPVQLG
EITEDDLKNYDGSDSKKPLLMAIKGQIYDVSQSRMFYGPGGPYALFAGKDASRALAKMSFEDKDLTGDISGL
GPFELEALQDWEYKFMSKYVKVGTIKKDAPPSDAPSPSEPSEAADVTDREAPKHAEDGPAETVEPSSVGDAE
KKEE*

>PB.16084.2 (CDS)

ATGCAATTAGTCGGGCTAAGAAATATATACGGGATGTTGGTGGAAGCCATACATGATTATTTCGGTCTG
TCTCCAACGGCATTCTTGGTCATTGTGGGCATAATGATAGTAACTTACAAGATTGTGTGTGGAATGTTTG
TGGCCGTCGATGATTACAAGGCAGTGAGAGATATGAAAGCGGTGTTTGAGGAGGATATGAGGCGGGA
GCCAGTGCAGCTGGGCGACGTAACGGAGGAGGAACTCAAGGCTTATGACGGGTCTGACCCAAAGAAG
CCGTTGCTGATGGCGATCAAGGGACAGATTTTCGACGTGTCCCGGGGCAGAATGTTCTACGGTCCTGGT
GGGCCCTATGCCATGTTTGCTGGTCATGATGCCACCAGAGCGCTAGCTCTTATGTCTTTTGATCCACAAG
ATCTAACCGGAAACACCGATGGCCTCAGTGAATCTGAAATAGACGTCCTCGAGGATTGGGAGCTCAAGT
TCAATGAGAAATATCCCAAGGTTGGCGTACTTGTATCCAAAGCTGCTATCGTCGACAATCAGCCTCTTGA
CACTAAAAAAGATGCTTAG

>PB.16084.2 (aa)

MQLVGLRNIYGMLVEAIHDYFGLSPTAFLVIVGIMIVTYKIVCGMFVAVDDYKAVRDMKAVFEEDMRREPV
QLGDVTEEELKAYDGSDPKKPLLMAIKGQIFDVSRGRMFYGPGGPYAMFAGHDATRALALMSFDPQDLTG
NTDGLSESEIDVLEDWELKFNEKYPKVGVLVSKAAIVDNQPLDTKKDA*

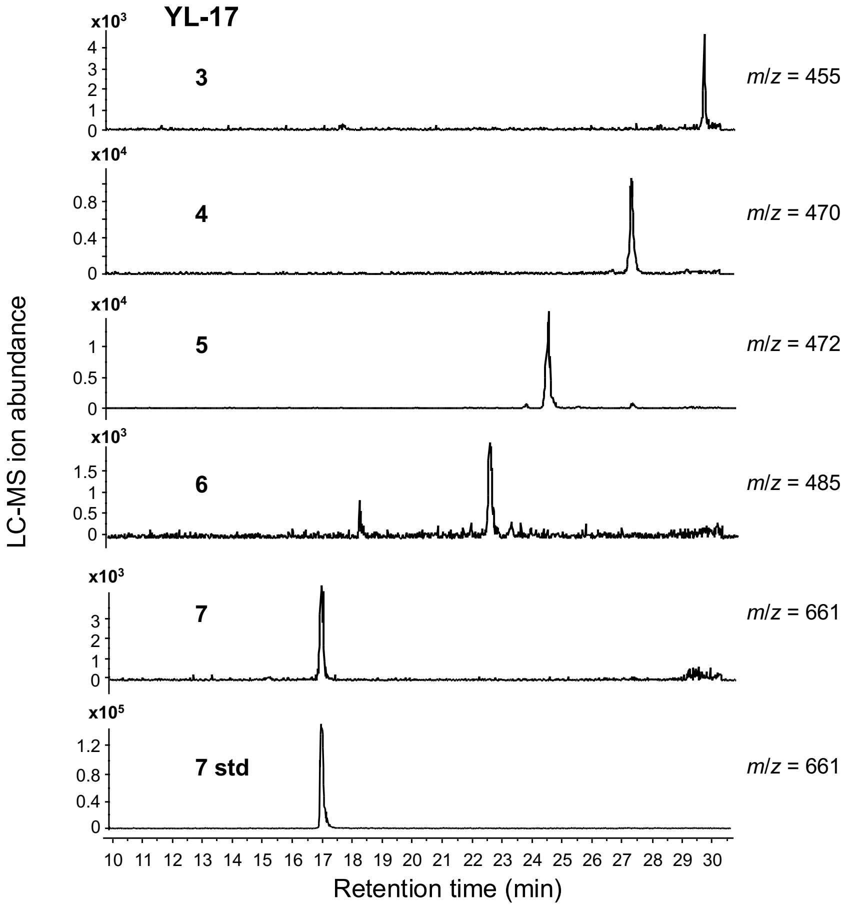

Supplementary Fig. 3 Extracted LC-MS ion chromatograms of quillaic acid (6) and C3-glucuronidated product 7. Co-expression of UGD and the C3 glucuronidating enzyme CLSM2 in YL-17 led to the formation of a new product, which was identified to be **7**.

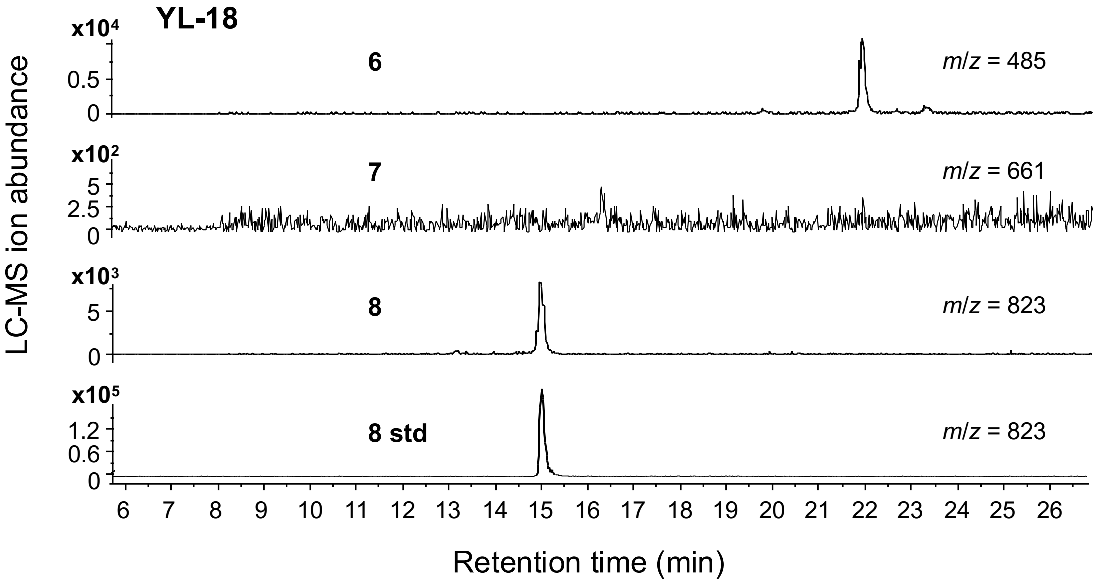

Supplementary Fig. 4 Extracted LC-MS ion chromatograms of quillaic acid (6) and C3 glycosylated products. Co-expression of UGD, the C3 glucuronidating enzyme CLSM2, as well as C3GalT in YL-18 led to the detection of the di-glycosylated product **8**, which co-eluted with the chemical standard. The galactosylation is an efficient step as no residual **7** was observed.

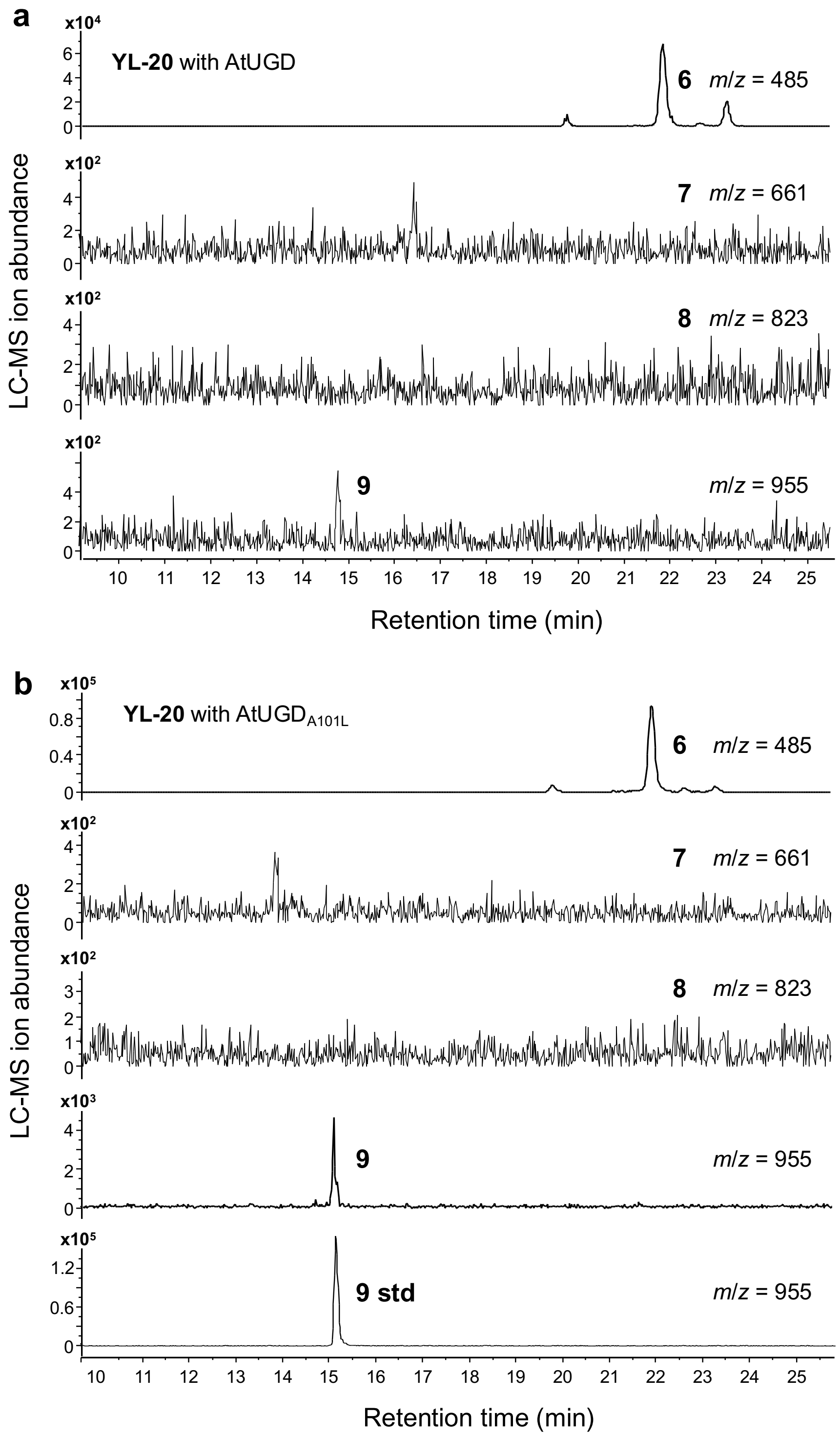

Supplementary Fig. 5 Extracted LC-MS ion chromatograms of quillaic acid and C3 glycosylated products. **a**, Due to the inhibition of UDP-Xyl on UDP-glucose dehydrogenase (UGD), no glycosylated products were observed when UDP-xylose synthase (UXS) was co-expressed. **b**, The $AtUGD_{A101L}$ mutant presumably has a lower binding affinity of UDP-Xyl, and thus, more tolerant

of UDP-Xyl, leading to the biosynthesis of the C3-fully glycosylated product (**9**). The biosynthesis of **9** was confirmed by the co-elution with the chemical standard.

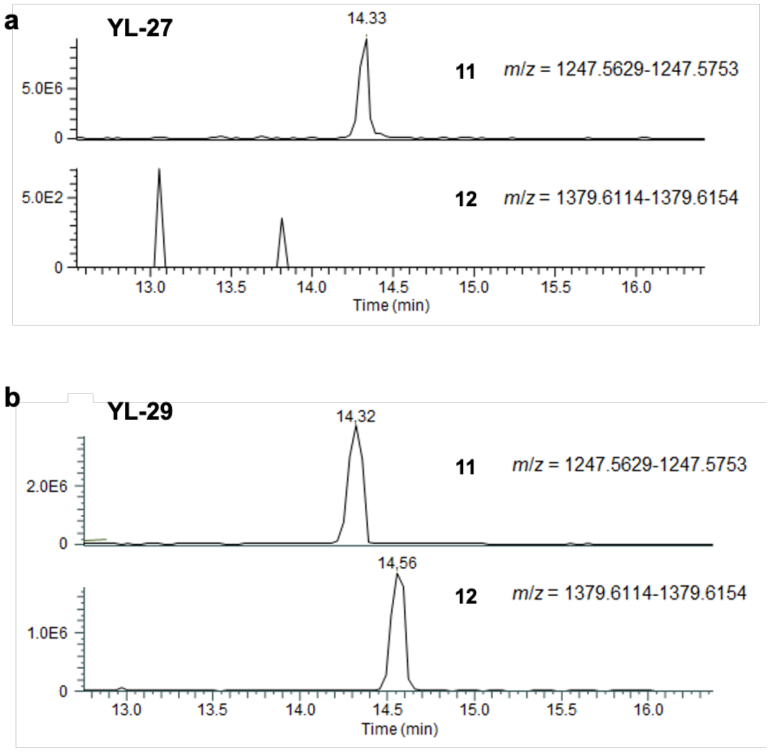

Supplementary Fig. 6 Extracted LC-MS ion chromatograms to confirm the production of C28-tri-glycosylated terpenoid, 12. Glycosylation of **11** produced by YL-27 (**a**) led to the formation of a new product with the high-resolution *m*/*z* of which corresponded to the molecular weight of **12** upon the expression of C28XylT3 in YL-29 shown in **b**.

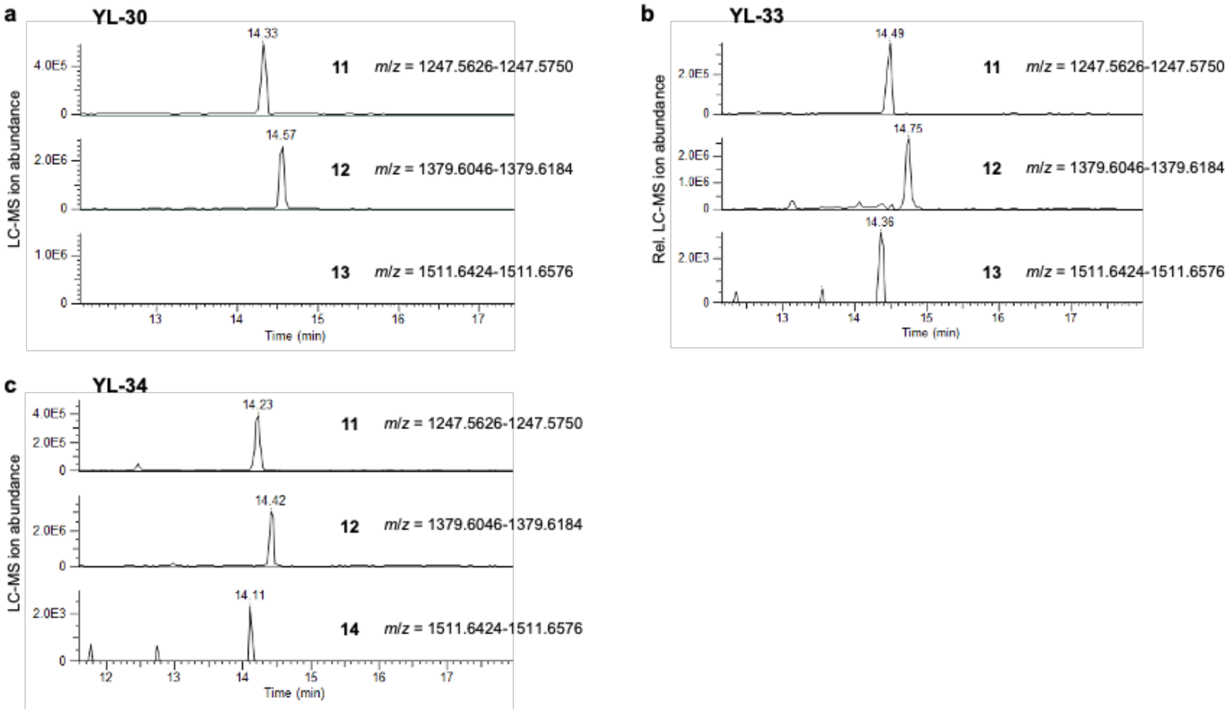

Supplementary Fig. 7 Extracted LC-MS ion chromatograms to confirm the production of C28 fully glycosylated 13 and 14. A new mass peak was detected that correspond to the C28-tetraglycosylated-molecule only in the presence of C28XylT4 in YL-33 (**b**) and C28ApiT in YL-34 (**c**), but not in the parent strain YL-30 (**a**).

Subcellular localization studies of UGT73CY3

Confocal images of cells with overexpressed C-terminally mCherry-tagged HSP42, which is a yeast native chaperone protein,[5,6] visualized its co-localization with the GFP-tagged protein aggregates (**Supplementary Fig. 8a**), but its overexpression only had a negligible impact on GT aggregation. Efforts towards solubilizing the GT, including N- and C-terminal truncation, solubility tagging (*e.g.*, SUMO, MBP, DsRED in YL-38 to YL-40, **Supplementary Fig. 8b**) yielded no noticeable improvements of its cytosolic expression. A time course study of the co-localization of RPN1, a subunit of the yeast proteasome, showed that upon nutrient depletion the proteasome initiates C28XylT4 degradation and recycling.[7,8] However, when fresh carbon and nitrogen resources are provided (*i.e.*, fresh YP galactose), the proteasome remains inactive and localized to the nucleus (**Supplementary Fig. 8c**). In addition, the expression of protein under galactose-inducible promoters is switched on when additional inducer (*i.e.*, galactose) is added to the media, leading to higher cytosolic expression of UGT73CY3. Extensive protein engineering efforts might be required to express fully functional and soluble C28XylT4 and C28ApiT4 in *Saccharomyces cerevisiae*.

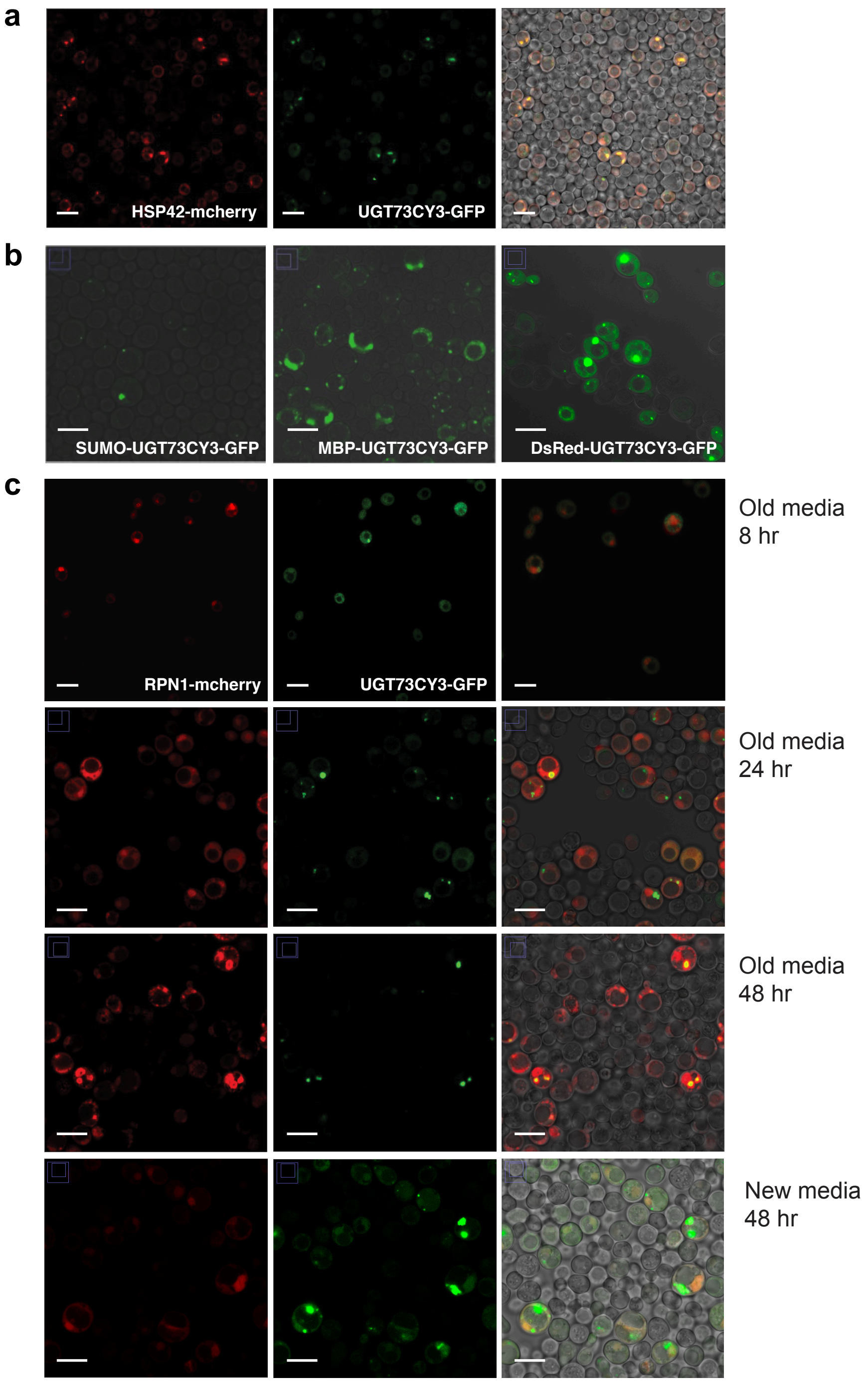

Supplementary Fig. 8 Subcellular localization studies in yeast of XylT4, solubility tagged XylT4, colocalization studies with HSP42 and RPN1. a, Confocal images of cells with overexpressed C-terminally mCherry-tagged HSP42, which is a yeast native chaperone protein visualized its co-localization with the GFP-tagged protein aggregates. **b**, Solubility tagging (*e.g.*, SUMO, MBP, DsRED yielded no noticeable improvements of the cytosolic expression of the GT. **c**, A time course study of the co-localization of RPN1, a subunit of the yeast proteasome, showed that upon nutrient depletion the proteasome initiates C28XylT4 degradation and recycling. However, when fresh carbon and nitrogen resources are provided (*i.e.*, fresh YP galactose), the proteasome remained inactive and

localized to the nucleus. In addition, the expression of protein under galactose-inducible promoters is switched on when additional inducer was added to the media, leading to higher cytosolic expression of the GT. Images were acquired using a Zeiss LSM 710 confocal microscope (scale bar represents 10 µm; at least three independent experiments were conducted).

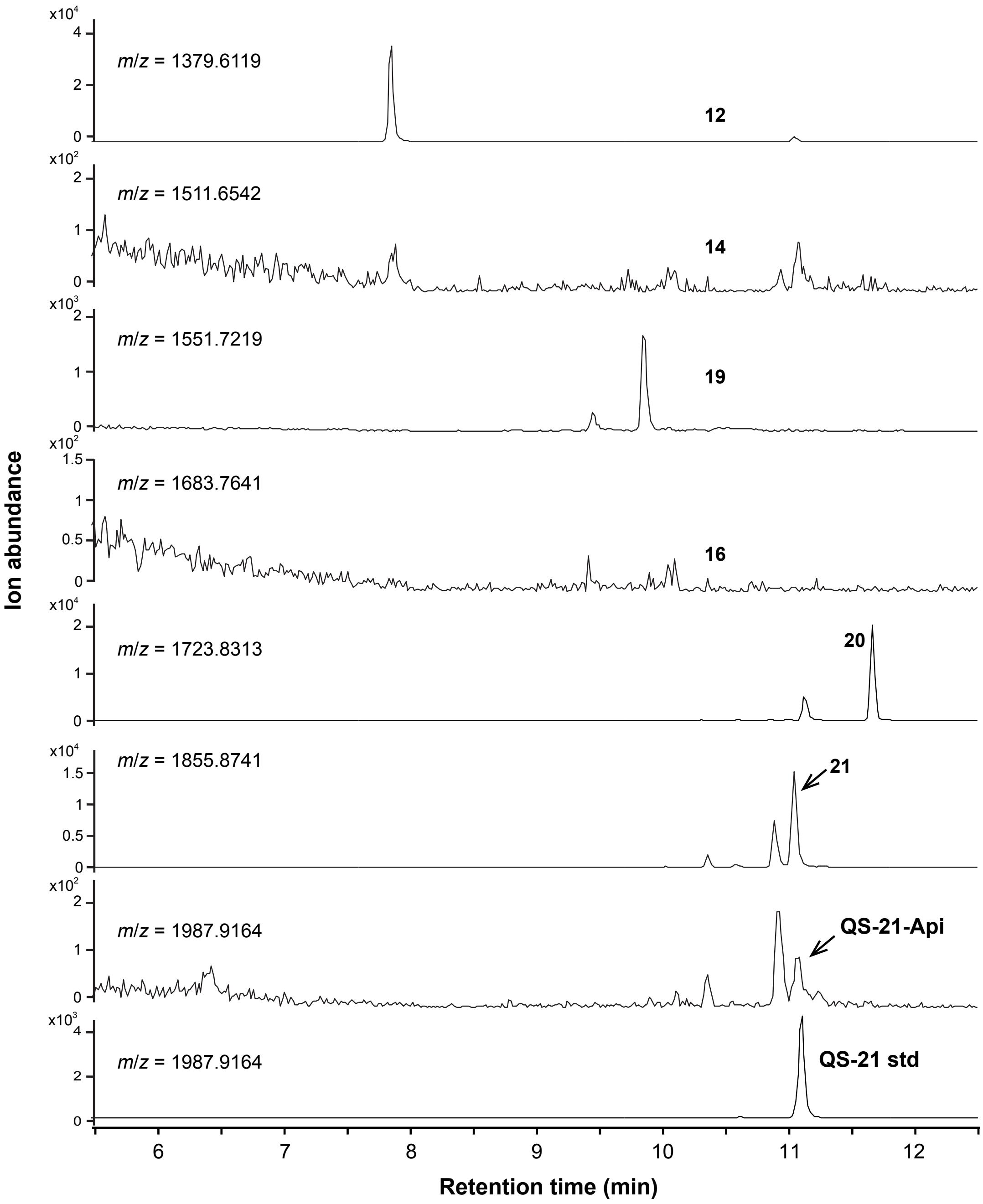

Supplementary Fig. 9 Production of QS-21-Api in the engineered yeast strain (YL-47). It was also observed from engineered yeasts that produce mono- and di-acylated as well as arabinofuranosylated C28-tri- and tetra-saccharides leading to the biosynthesis of QS-21-Api. The production of QS-21-Api was confirmed by its co-elution with the QS-21 standard. All LC-MS chromatograms were extracted with the theoretical m/z values of the respective compounds of interest.

QS-21 production and purification

YL-46 was cultured in 10 mL YPD medium in a 50-mL glass tube at 30 °C, 200 rpm for 16-18 hours as the seed culture. The seed culture was inoculated into 24-deep well plates with 2 mL YPD per well with an initial OD600 of 0.3. A total of ten of 24-deep well plates (approximately 500 mL) were cultured at 30 °C, 200 rpm following the same as the yeast strain culture condition described in the manuscript. A total of 53.8 µg QS-21 was produced and quantified using LCMS. The 24-deep well plates were centrifuged at 3000 g for 2 mins from which the supernatant was discarded and the cell pellets were washed with 500 µL H_2O per well and resuspended in 800 µL of MeOH/H2O (v:v=1:1) per well for QS-21 extraction by bead beating (3800 HZ, 3 mins). The cell pellets were extracted twice to yield a total of 41.9 µg of QS-21 in 200 mL of MeOH/H2O. Crude extracts from cell culture were fractionated by Reverse Phase-HPLC on an Agilent Prep-C18 column (5 µm, 21.2 x 150 mm) using a linear gradient of 0-45% acetonitrile (0.1% formic acid) in water (0.1% formic acid) over 4 min followed by 45-75% acetonitrile (0.1% formic acid) in water (0.1% formic acid) over 14 min at a flow rate of 4 mL/min, and finally followed by isocratic 75% acetonitrile (0.1% formic acid) in water (0.1% formic acid) for 3 min. The QS-21-Xyl peak area (tR19.6 min to 19.9 min) was collected and lyophilized. This crude QS-21 fraction was injected on an Agilent Zorbax Eclipse XDB-C18 semi-prep Column (5 µm, 9.4 x 250 mm) using a linear gradient of 30-50% over 18 min at a flow rate of 4 mL/min followed by isocratic 50% acetonitrile (0.1% formic acid) in water (0.1% formic acid) for 5 min. The QS-21-Xyl peak (tR = 19.9 min) was collected and lyophilized for MS and NMR analyses.

Tandem mass spectrometry

Samples and a QS-21 standard were analyzed using a liquid chromatography (LC) system (1200 series, Agilent Technologies, Santa Clara, CA) that was connected in line with a Thermo Exploris 120 mass spectrometer equipped with an electrospray ionization (ESI) source (Thermo Fisher Scientific, Waltham, MA) in negative ionization mode. LCMS method parameters are defined in **Supplementary Table 6**. The LC system contained the following modules: G1322A solvent degasser, G7120A binary pump, G1316C thermostatted column compartment, and G7167B autosampler unit (Agilent). Metabolites were separated using an Agilent 1290 series HPLC installed with a Kinetex 2.6 µm XB-C18 100 Å Column 50 x 2.1 mm column (00B-4496-AN, Phenomenex, Torrance, CA, USA). The MS data were acquired using an Orbitrap Exploris™ 120 Mass Spectrometer (Thermo Fisher Scientific, Waltham, MA, USA) in negative ionization mode. Raw files were converted to MZML using ProteoWizard MSConvert 3.0.21265-4230726.[14] Ion chromatograms and spectra were extracted using the RaMS package version 1.3.1 and plotted in base R 4.3.0[15]; vector-based PDFs were generated using Cairo 1.6. MS1 ion comparisons (SI x) are reported for the most intense ion across the EIC (with 5-ppm window). From a centroided mzml file, the most intense MS2 spectrum for each file was selected and then filtered to remove fragment ions <400 m/z and below 0.6% of the max intensity within each spectrum; a 15-ppm window was used to determine if sample fragment ions matched to standard reference compound fragment ions.

Supplementary Table 6. **LCMS method parameters used for QS-21 MS1 and MS2 measurement**

**Ion Source**	
Ion Source Type	H-ESI
Spray Voltage	Static
Negative Ion (V)	3000
Gas Mode	Static
Sheath Gas (Arb)	50
Aux Gas (Arb)	10
Sweep Gas (Arb)	2
Ion Transfer Tube Temp (C)	325
Vaporizer Temp (C)	50
Internal Mass calibration	Easy-IC run start
**FULL MS settings**	
resolution	120000
AGC target	Standard
Max. IT	100 ms
Scan range	700-2200 m/z
Spectrum data type	Profile
RT lens (%)	70
Microscans	1
**dd-MS2 settings**	
intensity threshold	2.00E+04
Dynamic exclusion	exclude after 1 time, 1 sec, exclude isotopes
Mass Tolerance (ppm)	0.1 (low); 5 (high)
Number of dependent scans	2
isolation window (m/z)	0.5
Normalized HCD collision energies (%)	13,30,80
resolution	60000
Scan Range Mode	Auto
AGC target	Standard
Max. IT	Auto
Microscans	10
Spectrum data type	Profile

**Target ion**	
m/z	z
1987.9133	1

Supplementary Table 7. MS2 fragment ion masses and their corresponding intensities

Retention time (min)	precursor m/z	fragment m/z	intensity	percent intensity	ppm difference	MS2 ion type
8.730585923	1987.912981	1988.916753	21407.15234	4.264490049	0.921671602	2nd isotope
8.730585923	1987.912981	1987.9072	501986.2188	100	0.553619908	fragment
8.730585923	1987.912981	1987.5341	3314.69458	0.660315853	0.306359043	fragment
8.730585923	1987.912981	1969.9118	25213.80664	5.022808535	1.082766731	fragment
8.730585923	1987.912981	1819.8519	4275.220703	0.851660971	-0.64001375	fragment
8.730585923	1987.912981	1725.7680	12801.62695	2.550194901	0.333040463	fragment
8.730585923	1987.912981	1665.7498	8558.392578	1.704905884	-0.79995268	fragment
8.730585923	1987.912981	1647.7350	24422.74219	4.865221649	0.081041332	fragment
8.730585923	1987.912981	1553.6701	88885.67188	17.70679524	-0.04390845	fragment
8.730585923	1987.912981	1511.6571	20978.5625	4.179111242	-0.45923246	fragment
8.730585923	1987.912981	955.4534	37817.30469	7.533534443	-1.30111594	fragment
8.730585923	1987.912981	485.3260	3084.617676	0.614482542	-1.46605919	fragment
8.730585923	1987.912981	469.1569	3207.034424	0.638869018	-2.99292551	fragment
8.730585923	1987.912981	405.3165	5145.628906	1.025053819	-2.16833423	fragment

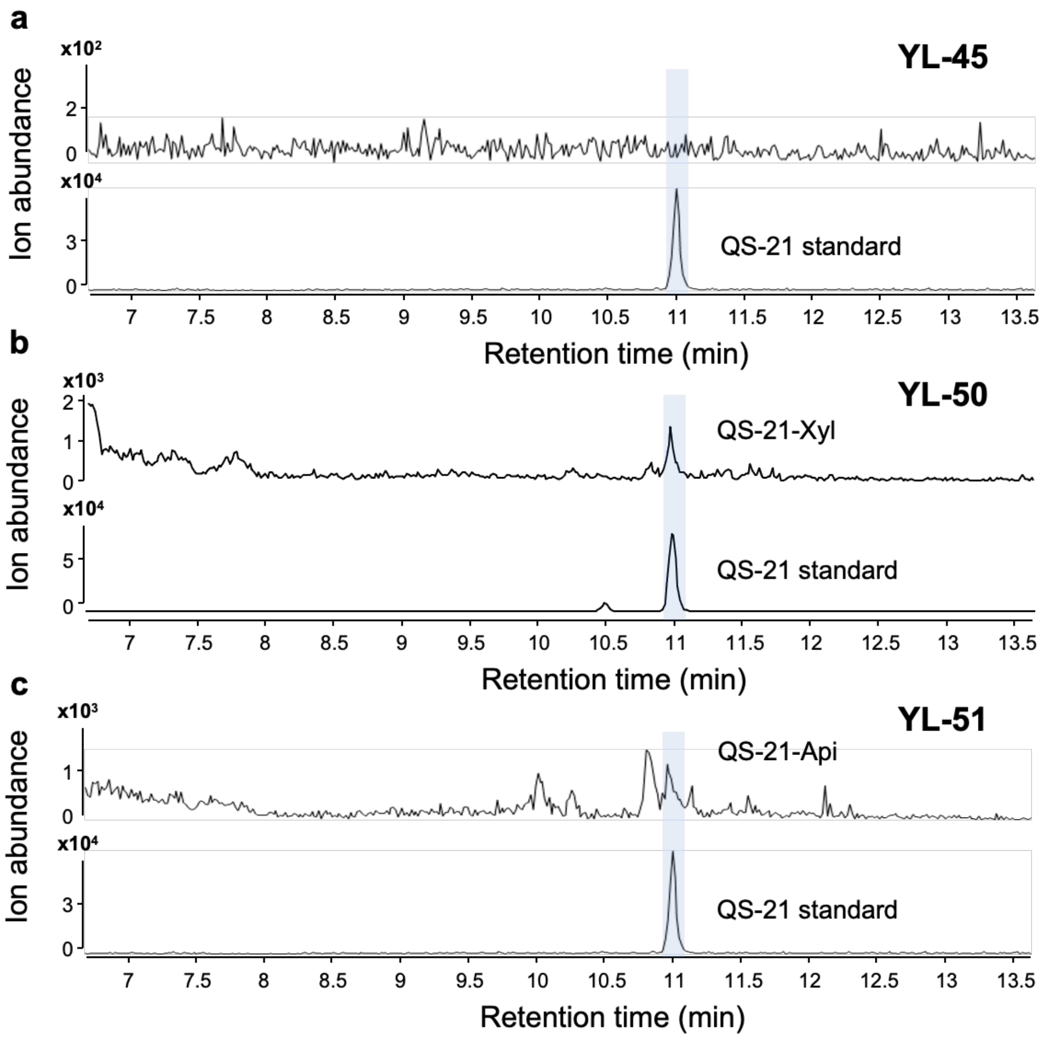

Supplementary Fig. 10 Total biosynthesis of QS-21 from simple sugars using engineered yeast strains compared to the QS-21 standard. a, No QS-21 was detected in the background strain YL-45. **b**, QS-21-Xyl produced by engineered yeast YL-50. **c**, QS-21-Api produced by engineered yeast YL-51. All LC–MS chromatograms were extracted with the theoretical m/z values of the respective compounds of interest.

Calculations of the QS-21 content and production rate in *Q. saponaria* and *S. cerevisiae*

Q. saponaria: The production of QS-21 mainly is sourced from the bark, which is approximately 20% of the tree mass. Of bark, extraction and dialysis led to a crude extract DQ.[9] Further purification of DQ led to the saponin mixture Quil A (250 mg from the pool of 6 batches of 5 g of bark,[9,10] 0.8% yield from the bark). From Quil A, the purification of QS-21 was recently reported to be approximately 2%.[11,12]

The overall QS-21 yield from *Q. saponaria*:

$$20\% * \frac{250\,mg}{5\,g * 6} * 2\% = 0.0032\%$$

Quillaja saponaria produces QS-21 after 30-50 years[17] and a maximum of 35% of the basal tree area can be extracted every 5-10 years from the same site, enforced by the forestry management plan has been approved by CONAE[17], which makes it rate of production:

$$\frac{0.0032\%}{10\,years}\frac{1\,year}{365\,days} * 35\% = 3 \cdot 10^{-7}\%/\text{day}$$

S. cerevisiae: Yeast dry cell weight is approximately 7.04 g L^{-1} which can be calculated based on the OD of 25 divided by the index of OD conversion to dry cell weight (25/3.55 = 7.04 g L^{-1}).[13] The titer of QS-21 in YL-46 is 0.9 mg L^{-1}, which led to the QS-21 content in yeast to be:

$$\frac{0.09\,\frac{mg}{L}}{25 * \frac{1\,g\,DCW}{3.55\,L}} = 0.0012\%$$

S. cerevisiae produces that amount over a period of approximately 4 days, which makes it rate of production:

$$\frac{0.0012\%}{4\,days} = 3 \cdot 10^{-4}\%/\text{day}$$

Hence, unoptimized *S. cerevisiae* produces QS-21-xyl at approximately 1,000 times the rate of *Q. saponaria*.

References

1. Reed, J. *et al.* Elucidation of the pathway for biosynthesis of saponin adjuvants from the soapbark tree. *Science*, **379**, 1252-1264 (2023).
2. Chen, X. *et al.* Deciphering triterpenoid saponin biosynthesis by leveraging transcriptome response to Methyl Jasmonate elicitation in *Saponaria vaccaria*. *Nat. Comm*. **14**, 7101 (2023).
3. Martin, L.B.B. *et al*. Complete biosynthesis of the potent vaccine adjuvant QS-21. *Nat. Chem. Biol*. *Accepted.*
4. Wong, J., d'Espaux, L., Dev, I., van der Horst, C. & Keasling, J. *De novo* synthesis of the sedative valerenic acid in *Saccharomyces cerevisiae*. *Metab. Eng.* **47**, 94–101 (2018).
5. Sathyanarayanan, U. *et al.* ATP hydrolysis by yeast Hsp104 determines protein aggregate dissolution and size in vivo. *Nat. Commun.* **11**, 5226 (2020).
6. Lee, H.-Y., Chao, J.-C., Cheng, K.-Y. & Leu, J.-Y. Misfolding-prone proteins are reversibly sequestered to an Hsp42-associated granule upon chronological aging. *J. Cell Sci.* **131**, jcs.220202 (2018).
7. van Deventer, S., Menendez-Benito, V., van Leeuwen, F. & Neefjes, J. N-terminal acetylation and replicative age affect proteasome localization and cell fitness during aging. *J. Cell Sci.* **128**, 109–117 (2015).
8. Enenkel, C., Kang, R. W., Wilfling, F. & Ernst, O. P. Intracellular localization of the proteasome in response to stress conditions. *J. Biol. Chem.* **298**, 102083 (2022).
9. Dalsgaard, K. Saponin adjuvants. I. The presence of a non-dialysable fraction of *Quillaja saponaria* Molina with adjuvant activity in foot-and-mouth disease vaccines. *Bull. Off. int. Epiz.* **77**, 1289-1295 (1972).
10. Dalsgaard, K. Saponin adjuvants III. Isolation of a substance from *Quillaja saponaria* molina with adjuvant activity in foot-and-mouth disease vaccines. *Archiv fir die gesamte Virusforschung* **44**, 243-254 (1974).
11. *Saponins Used in Traditional and Modern Medicine*, Springer New York, NY (1996).
12. Qi, Y. & Fox, C. B. A Two-step orthogonal chromatographic process for purifying the molecular adjuvant QS-21 with high purity and yield. *J. Chromatogr. A* **1635**, 461705 (2021).
13. Garay-Arroyo, A. *et al*. Response to different environmental stress conditions of industrial and laboratory *Saccharomyces cerevisiae* strains. *Appl. Microbiol. Biotechnol.* **63**, 734–741 (2004).
14. Chambers, *et al.* A cross-platform toolkit for mass spectrometry and proteomics. Nat. Biotechnol. **30**, 918-920 (2012).
15. R Core Team (2023). _R: A Language and Environment for Statistical Computing_. R Foundation for Statistical Computing, Vienna, Austria. <https://www.R-project.org/>.
16. Wallace, F., Fontana, C., Ferreira, F., Olivaro, C. Structure elucidation of triterpenoid saponins found in an immunoadjuvant preparation of *Quillaja brasiliensis* using mass spectrometry and ^{1}H and ^{13}C NMR spectroscopy. *Molecules* **27**, 2402 (2022).
17. San Martin, R., Briones, R. Industrial uses and sustainable supply of *Quillaja saponaria* (Rosaceae) saponins. *Econ. Bot.* **53**, 302–311 (1999).
