# Supplementary material for: Complete biosynthesis of QS-21 in engineered yeast
Source: Nature. 2024 May 8;629(8013):937–44. doi: 10.1038/s41586-024-07345-9 (PMC11111400; doi:10.1038/s41586-024-07345-9)
Supplement: Supplementary file 2 — Reporting Summary [file 41586_2024_7345_MOESM2_ESM.pdf]

nature portfolio

Corresponding author(s): Jay D. Keasling

Last updated by author(s): Feb 24, 2024

Reporting Summary

Nature Portfolio wishes to improve the reproducibility of the work that we publish. This form provides structure for consistency and transparency in reporting. For further information on Nature Portfolio policies, see our Editorial Policies and the Editorial Policy Checklist.

Statistics

For all statistical analyses, confirm that the following items are present in the figure legend, table legend, main text, or Methods section.

n/a	Confirmed	
☐	☒	The exact sample size (*n*) for each experimental group/condition, given as a discrete number and unit of measurement
☐	☒	A statement on whether measurements were taken from distinct samples or whether the same sample was measured repeatedly
☐	☒	The statistical test(s) used AND whether they are one- or two-sided<br>*Only common tests should be described solely by name; describe more complex techniques in the Methods section.*
☒	☐	A description of all covariates tested
☒	☐	A description of any assumptions or corrections, such as tests of normality and adjustment for multiple comparisons
☐	☒	A full description of the statistical parameters including central tendency (e.g. means) or other basic estimates (e.g. regression coefficient) AND variation (e.g. standard deviation) or associated estimates of uncertainty (e.g. confidence intervals)
☐	☒	For null hypothesis testing, the test statistic (e.g. *F*, *t*, *r*) with confidence intervals, effect sizes, degrees of freedom and *P* value noted<br>*Give P values as exact values whenever suitable.*
☒	☐	For Bayesian analysis, information on the choice of priors and Markov chain Monte Carlo settings
☒	☐	For hierarchical and complex designs, identification of the appropriate level for tests and full reporting of outcomes
☒	☐	Estimates of effect sizes (e.g. Cohen's *d*, Pearson's *r*), indicating how they were calculated

Our web collection on statistics for biologists contains articles on many of the points above.

Software and code

Policy information about availability of computer code

Data collection	LC-MS data were acquired using MassHunter Workstation Data Acquisition Software version B.08.00 (for high resolution mass and MS/MS) or Agilent OpenLab CDS version 2.4 were used for data acquisition.
Data analysis	LC-MS data were analyzed using Agilent MassHunter Qualitative Analysis version B.06.00 (for high resolution mass and MS/MS) or Agilent OpenLab CDS version 2.4 were used for data analysis.

For manuscripts utilizing custom algorithms or software that are central to the research but not yet described in published literature, software must be made available to editors and reviewers. We strongly encourage code deposition in a community repository (e.g. GitHub). See the Nature Portfolio guidelines for submitting code & software for further information.

Data

Policy information about availability of data

All manuscripts must include a data availability statement. This statement should provide the following information, where applicable:

- Accession codes, unique identifiers, or web links for publicly available datasets
- A description of any restrictions on data availability
- For clinical datasets or third party data, please ensure that the statement adheres to our policy

Nucleotide sequence data of Saponaria vaccaria MSBPs are available in the third-party annotation section of the DDBJ/ENA/GenBank databases (Supplementary Table 1). Strains and plasmids developed for this study (Supplementary Table 2), along with annotated sequences, have been deposited in the JBEI Registry (https://

registry.jbei.org) and are physically available from the authors upon reasonable request. Contractual obligations from commercial partnerships prohibit us from distributing (by ourselves or through a third party) strains described in our manuscript to for-profit commercial entities. However, we provide extensive genotypic descriptions of our strains, fully annotated DNA sequences, and detailed methods that enable others to build upon our work. Strains will be provided to nonprofit, government, or academic laboratories and institutions.

Research involving human participants, their data, or biological material

Policy information about studies with human participants or human data. See also policy information about sex, gender (identity/presentation), and sexual orientation and race, ethnicity and racism.

Reporting on sex and gender	N/A
Reporting on race, ethnicity, or other socially relevant groupings	N/A
Population characteristics	N/A
Recruitment	N/A
Ethics oversight	N/A

Note that full information on the approval of the study protocol must also be provided in the manuscript.

Field-specific reporting

Please select the one below that is the best fit for your research. If you are not sure, read the appropriate sections before making your selection.

☒ Life sciences ☐ Behavioural & social sciences ☐ Ecological, evolutionary & environmental sciences

For a reference copy of the document with all sections, see nature.com/documents/nr-reporting-summary-flat.pdf

Life sciences study design

All studies must disclose on these points even when the disclosure is negative.

Sample size	Sample size of three or more was taken following previous papers in this field.
Data exclusions	No data were excluded in the processing of data analysis.
Replication	All experiments were performed in triplicates or more. All attempts at replication were successful.
Randomization	Replicates of all engineered strains were randomly picked up from corresponding selection plates for data generation.
Blinding	No blinding was performed, as no subjective measurements were done.

Reporting for specific materials, systems and methods

We require information from authors about some types of materials, experimental systems and methods used in many studies. Here, indicate whether each material, system or method listed is relevant to your study. If you are not sure if a list item applies to your research, read the appropriate section before selecting a response.

Materials & experimental systems

n/a	Involved in the study	
☒	☐	Antibodies
☐	☒	Eukaryotic cell lines
☒	☐	Palaeontology and archaeology
☒	☐	Animals and other organisms
☒	☐	Clinical data
☒	☐	Dual use research of concern
☒	☐	Plants

Methods

n/a	Involved in the study	
☒	☐	ChIP-seq
☒	☐	Flow cytometry
☒	☐	MRI-based neuroimaging

Eukaryotic cell lines

Policy information about cell lines and Sex and Gender in Research

Cell line source(s)	All strains used in this study were derived from the Saccharomyces cerevisiae strain CEN.PK2-1C (EuroSCARF3 0000A).
Authentication	All yeast strains with chromosomal editing were validated by genotyping PCR and sequencing of the modified loci.
Mycoplasma contamination	N/A
Commonly misidentified lines (See ICLAC register)	N/A

Plants

Seed stocks	N/A
Novel plant genotypes	N/A
Authentication	N/A
